# Mathematical modelling of breast cancer cells in response to endocrine therapy and Cdk4/6 inhibition

**DOI:** 10.1098/rsif.2020.0339

**Published:** 2020-08-26

**Authors:** Wei He, Diane M. Demas, Isabel P. Conde, Ayesha N. Shajahan-Haq, William T. Baumann

**Affiliations:** 1 Program in Genetics, Bioinformatics, and Computational Biology, VT BIOTRANS, Virginia Tech, Blacksburg, VA, USA; 2 Department of Oncology, Lombardi Comprehensive Cancer Center, Georgetown University Medical Center, Washington, DC, USA; 3 Department of Electrical and Computer Engineering, Virginia Tech, Blacksburg, VA, USA

**Keywords:** mathematical modelling, breast cancer, therapy optimization, endocrine therapy, palbociclib

## Abstract

Oestrogen receptor (ER)-positive breast cancer is responsive to a number of targeted therapies used clinically. Unfortunately, the continuous application of any targeted therapy often results in resistance to the therapy. Our ultimate goal is to use mathematical modelling to optimize alternating therapies that not only decrease proliferation but also stave off resistance. Toward this end, we measured levels of key proteins and proliferation over a 7-day time course in ER+ MCF-7 breast cancer cells. Treatments included endocrine therapy, either oestrogen deprivation, which mimics the effects of an aromatase inhibitor, or fulvestrant, an ER degrader. These data were used to calibrate a mathematical model based on key interactions between ER signalling and the cell cycle. We show that the calibrated model is capable of predicting the combination treatment of fulvestrant and oestrogen deprivation. Further, we show that we can add a new drug, palbociclib, to the model by measuring only two key proteins, cMyc and hyperphosphorylated RB1, and adjusting only parameters associated with the drug. The model is then able to predict the combination treatment of oestrogen deprivation and palbociclib. We illustrate the model's potential to explore protocols that limit proliferation and hold off resistance by not depending on any one therapy.

## 1. Introduction

Despite advances in treatment options, it is estimated that 42 260 women and men will die as a result of breast cancer in the USA this year [1]. Seventy per cent of all breast cancer cases are oestrogen receptor positive (ER+) and targeted therapies such as endocrine therapy or Cdk4/6 inhibitors are used to treat this clinical subtype, resulting in dramatic improvements in long-term survival rates. Endocrine therapies act to decrease ER signalling in a variety of ways: (i) by depriving the ER of its ligand oestrogen (17β-oestradiol, hereafter referred to as E2) via aromatase inhibitors (e.g. letrozole), (ii) by competitively inhibiting the binding of E2 to the ER (the mode of action of tamoxifen), or (iii) by increasing the degradation of the ER via the proteasome (the mode of action of ICI 182 780 (ICI; Faslodex/fulvestrant)). This inhibition of ER signalling can halt proliferation by arresting cells in G1 and inducing cell death [2–4]. Inhibitors of cyclin-dependent kinases Cdk4/6, such as palbociclib, have been used in conjunction with endocrine therapies to provide a more durable inhibition of proliferation [5–7].

Unfortunately, long-term application of a targeted therapy may lead to the development of resistance and recurrence of cancer [8–12]. However, sequential application of various therapies and drug holidays, in a repeating cycle, may provide a means of delaying resistance. To develop such a sequential therapy, in light of the many possibilities for drugs, doses and timings, mathematical modelling of the system is likely to be critical [13]. Ultimately, such a model should be able to incorporate numerous drugs, predict the proliferation of ER+ cells in response to these drugs over relatively long time scales and account for some of the many possible resistance mechanisms. Such a model would allow for optimizing sequential therapies, subject to numerous constraints, to propose therapeutic protocols that can be evaluated experimentally.

Our study takes the first step along this road by building a model, based on known mechanisms from the literature, to describe the changes in protein expression and proliferation of an E2-dependent, asynchronous population of ER+ MCF-7 breast cancer cells in response to various clinically relevant therapies over a time period of one week. The therapies we consider are E2 deprivation (a surrogate for aromatase inhibitors), ICI (a selective ER degrader) and palbociclib (a Cdk4/6 inhibitor). Because the model has a mechanistic basis, we show that it is relatively straightforward to add additional drugs relevant to the mechanism and to predict the effects of combinations of drugs. We also show how the model might be used to develop protocols that delay resistance, once it has been validated at longer time scales and the dynamics of resistance acquisition are better understood.

While there have been a number of previous mathematical models dealing with ER signalling and its impact on the cell cycle [14–16], these models differ from ours in that they are phenomenological and not experimentally calibrated or validated. There are also a number of mathematical models that describe the G1–S transition in single cells [17–21]. Most of these investigate the bistable switch governing the commitment to proliferation at the restriction point [17]. While our model is based on a similar structure, our aim is to capture the behaviour of an asynchronous population of cells, creating an average model, so we do not expect our parameterization to create a bistable switch or be valid for single cells.

Before presenting our results, it is important to clarify what can be expected of a model of the type we propose. The behaviour of protein levels in cells in response to therapeutic perturbations is the result of thousands, if not millions, of interactions among DNA, RNA and proteins. It is clearly impossible at present to model this level of complexity, and so of necessity we use a vastly simplified model to attempt to capture the key effects of therapy. This has two important implications for the modelling. First, many reaction rates used in the model account for numerous unmodelled effects (e.g. the rate at which ligand-bound ER upregulates the transcription factor c-Myc), and thus there is no corresponding rate in the literature. Second, the simple model is unlikely to provide an excellent match to the experimental data. Therefore, our goal is to produce a model that fits the data as well as possible and then show that the model is useful for its intended purpose: predicting the results of combination therapies and making it easy to add new drugs that operate on the mechanisms being modelled.

## 2. Material and methods

### 2.1. Cell culture and reagents

MCF-7 cells were obtained from Tissue Culture Shared Resources at Lombardi Comprehensive Cancer Center, Georgetown University, Washington, DC [22,23]. MCF-7 cells depend on E2 for growth, and therefore, to enable us to control the level of E2 in the cell culture medium, cells were grown in phenol red-free improved minimal essential medium (Life Technologies, Grand Island, NY; A10488-01) with 10% charcoal-stripped calf serum (CCS) and supplemented with 10 nM 17β-oestradiol (oestrogen; E2). E2 deprivation was obtained by washing cells 24 h post-plating (*t* = 0) with phosphate-buffered saline (PBS) and adding complete medium without E2 for the indicated times. ICI (Faslodex/Fulvestrant; ICI182,780) and palbociclib were obtained from Tocris Bioscience (Ellisville, MO). MCF-7 cells were authenticated by DNA fingerprinting and tested regularly for *Mycoplasma* infection. All other chemicals were purchased from Sigma-Aldrich (St. Louis, MO).

### 2.2. Cell proliferation assays

Cells were seeded at a density of 1–2 × 10^5^ cells well^−1^ or 4–5 × 10^4^ cells well^−1^ in 100 mm or 60 mm plates, respectively. Cells were then trypsinized, resuspended in PBS and counted using a Z1 Single Coulter Counter (Beckman Coulter, Miami, FL). Three independent experiments were done. Data were normalized to cell number at *t* = 0 and are presented as the mean ± s.e. from all three experiments.

### 2.3. Western blot analysis

For Western blot analysis, cells were lysed for 30 min at 4°C in lysis buffer (50 mM Tris-HCl, pH 7.5, containing 150 mM NaCl, 1 mM EDTA, 0.5% sodium deoxycholate, 1% IGEPAL CA-630, 0.1% sodium dodecyl sulfate (SDS), 1 mM Na_3_VO_4_, 44 µg ml^−1^ phenylmethylsulfonyl fluoride) supplemented with Complete Mini protease inhibitor mixture tablets (Roche Applied Science). Total protein was quantified using the bicinchoninic acid assay (Pierce). Whole-cell lysate (20 µg) was resolved by SDS–polyacrylaminde gel electrophoresis. The following antibodies were used: c-Myc (no. 5605), cyclinD1 (no. 2978), p21 (no. 2947) and RB1 (no. 9309) were from Cell Signaling, Danvers, MA; ESR*α* (MA5-14104) was from Invitrogen; RB1-phosphorylated on Ser612 (OAAB16108) was from Aviva Systems Biology, San Diego, CA; actin (sc-47778) was from Santa Cruz Biotechnology, Santa Cruz, CA; β-tubulin (T7816) was from Sigma, St. Louis, MO.

### 2.4. Data collection

Table 1 summarizes the data that were collected. Protein levels were analysed by western blotting and cell numbers were determined using a Coulter counter. Protein levels and cell numbers were first normalized to 0 h. Protein levels in treatment conditions were then normalized to the +E2 case (control; untreated, grown with E2 in medium). See the electronic supplementary material for details.

**Table 1.** Time points at which the various protein species and cell numbers were measured in response to the various treatments. +E2 (untreated, grown with E2 in medium), +E2 + ICI (treated with ICI in +E2 medium), –E2 (deprived of E2), –E2 + ICI (treated with ICI in –E2 medium), +E2 + palbociclib (treated with palbociclib in +E2 medium) and –E2 + palbociclib (treated with palbociclib in –E2 medium). Underlined time points were used for normalization; italicized for model parameter calibration; and bolded for model validation. h: hours and d: days.

condition	protein name	protein time points	cell number time points
+E2 (control)	ER, c-Myc, cyclinD1, p21, RB1-pp, total RB1	0 h, 4 h, 1d, 3d, 6d, 7d	0 h, *1d*, *3d*, *6d*, *7d*
+E2 + ICI	ER, c-Myc, cyclinD1, p21, RB1-pp, total RB1	0 h, *4 h*, *1d*, *3d*, *6d*, *7d*	0 h, *1d*, *3d*, *6d*, *7d*
–E2	ER, c-Myc, cyclinD1, p21, RB1-pp, total RB1	0 h, *4 h*, *1d*, *3d*, *6d*, *7d*	0 h, *1d*, *3d*, *6d*, *7d*
–E2 + ICI	ER, c-Myc, cyclinD1, p21, RB1-pp, total RB1	0 h, **4 h**, **1d**, **3d**, **6d**, **7d**	0 h, **1d**, **3d**, **6d**, **7d**
+E2 + palbo	c-Myc, RB1-pp	0 h, *1d*, *3d*, *6d*, *7d*	0 h, *1d*, *3d*, *6d*, *7d*
–E2 + palbo	c-Myc, RB1-pp	0 h, **1d**, **3d**, **6d**, **7d**	0 h, **1d**, **3d**, **6d**, **7d**

### 2.5. Model structure

**Figure 1.**
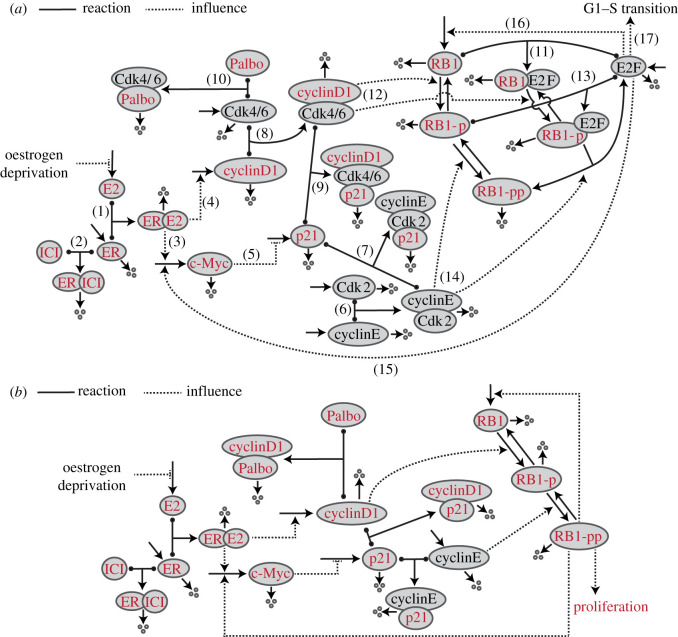
Wiring diagrams of the biological mechanism and the mathematical model. Solid lines with balls represent reversible binding reactions, other solid lines represent production or degradation of proteins, dashed lines represent influences with arrowheads representing enhancement and blunt heads representing inhibition. (*a*) Biological mechanism consisting of the following processes: (1) ER binds its ligand E2; (2) ICI binds ER and enhances its degradation; (3) ER:E2 enhances production of c-Myc; (4) ER:E2 enhances production of cyclinD1; (5) c-Myc inhibits the production of p21; (6) cyclinE binds to Cdk2; (7) p21 binds to cyclinE:Cdk2 and inactivates it; (8) cyclinD1 binds to Cdk4/6; (9) p21 binds to cyclinD1:Cdk4/6 and inactivates it; (10) palbociclib binds to Cdk4/6 and inactivates it; (11) RB1 binds to E2F inactivating it; (12) cyclinD1:Cdk4/6 phosphorylates RB1; (13) RB1-p binds to E2F; (14) cyclinE:Cdk2 phosphorylates RB1-p, preventing it from binding E2F; (15) free E2F enhances production of c-Myc; (16) free E2F enhances production of RB1; (17) free E2F drives the G1–S transition and proliferation. (*b*) Mathematial model wiring diagram. The species in red were measured by experiment and those in black were not. For p21, cyclinD1 and RB1, the total protein was measured. In addition, RB1-pp was measured.

The interactions of key elements governing the biological mechanism associated with the effect of E2 signalling on proliferation are shown in figure 1*a*. E2 bound to ER enhances the production of cyclinD1 and c-Myc. CyclinD1 with its kinase partner Cdk4/6 drives RB1 to a hypophosphorylated state, RB1-p. CyclinE with its kinase partner Cdk2 drives RB1-p to a hyperphosphorylated state, RB1-pp, in which it can no longer bind and inhibit E2F, allowing E2F to drive the G1–S transition and proliferation. p21, representing both itself and p27, inactivates both cyclinD1:Cdk4/6 and cyclinE:Cdk2, but the increased production of c-Myc increases its inhibition of p21 production, helping to take the brakes off proliferation. Further details and references are provided in the electronic supplementary material.

In the mathematical model (figure 1*b*), we make some simplifying assumptions to reduce the number of species modelled so as to be more in line with the number of species measured. In particular, we do not model Cdk4/6 explicitly, but assume that all cyclinD1 not bound to p21 is bound to Cdk4/6 and active. Thus, to model the effect of palbociclib, which inactivates cyclinD1:Cdk4/6, we allow palbociclib to bind to cyclinD1 in the model and hold it inactive. Similarly, we do not explicitly model Cdk2. In addition, we do not model E2F, but assume that the level of RB1-pp reflects the transcriptional activity of E2F. While not modelling E2F may seem a step too far, the actual biological complexity is more significant than that shown in figure 1*a*. There are three pocket proteins (RB1, p107 and p130) that bind to the nine different E2F species (E2F1–9), and the binding efficiencies are governed by many phosphorylation sites on the pocket proteins [24]. Since our ultimate objective is predicting proliferation, we settled on associating the RB1-pp level (associated with phosphorylation on S612, which is associated with proliferation [25]) with proliferation, as this matches our experimental data.

### 2.6. Modelling oestrogen deprivation

Removing E2 from cultured cells that have been growing in medium containing E2 cannot be accomplished by simply changing to a medium containing no E2 [26]. MCF-7 cells growing in +E2 conditions have a much higher internal concentration of E2 than that of the medium due to non-specific binding of E2 in the cytoplasm as well as specific binding of E2 to various oestrogen receptors in the cell. When the medium is changed to a –E2 medium, E2 from the cells leaches into the new medium, establishing a new E2 level that can be significant for maintaining proliferation as MCF-7 cells can proliferate significantly in 10 pM of E2 [27]. In our 7-day experiments, the medium was changed to treated medium on day 0 and on day 3. For treatments involving E2 deprivation, we incorporated two additional model parameters, one for the E2 concentration in the medium after day 0 and one for the concentration after day 3, to account for the fact that the E2 concentration does not go to zero and that it decreases with additional changes in the medium.

### 2.7. Parameter estimation

The degradation rates for different proteins were assigned according to half-lives found in the literature, where *k_d_* = −log(1/2)/*T*_half-life_. The other parameters were optimized to minimize the discrepancy between the model simulation and the experimental result. The cost function was

$$M(p)=\overset{n}{\underset{i=1}{\sum }}\overset{m}{\underset{\;j=1}{\sum }}\frac{{\left(y_{ij}^{E}(t_{j})-y_{ij}(t_{\;j,}p)\right)}^{2}}{\sigma_{ij}^{2}},$$

where *i* indexes the state variables (proteins or cell number), *j* indexes the experimental time points, $y_{ij}^{E}(t_{j})$ is the experimental measurement of the *i*th variable at time *j*, *y_ij_*(*t_j_*, *p*) is the simulation result of the *i*th measurement at time *j* using parameter vector *p*, and *σ_ij_* is the standard deviation of the measured value based on three replications. The data and time points used for parameter estimation are listed in table 1. Parameter estimation was performed using MATLAB [28]. Initially, we tuned the model manually to get the gross responses correct. Then we relied on the default genetic algorithm function, *ga,* in MATLAB's global optimization toolbox and used the *fminsearch* function to refine the results of *ga*. We broke the parameters into groups, initially tuning the parameters related to ER, then tuning those related to all other proteins, and finally tuning all these parameters together. The three parameters associated with palbociclib were tuned separately after the initial model was tuned. The parameters associated with proliferation were tuned last, and finally all the parameters were tuned together to produce the final result.

### 2.8. Statistical analysis

The Mann–Whitney U-test was used for statistical comparisons [29]. The western blot protein data from the treatment conditions were first normalized to the control condition and then compared with 1 to test whether the treatments significantly decrease or increase the protein levels after time 0. Cell numbers after treatment were directly compared with the control numbers to test whether the treatments decreased cell proliferation.

## 3. Results

### 3.1. The proposed model structure can largely explain the experimental data

**Figure 2.**
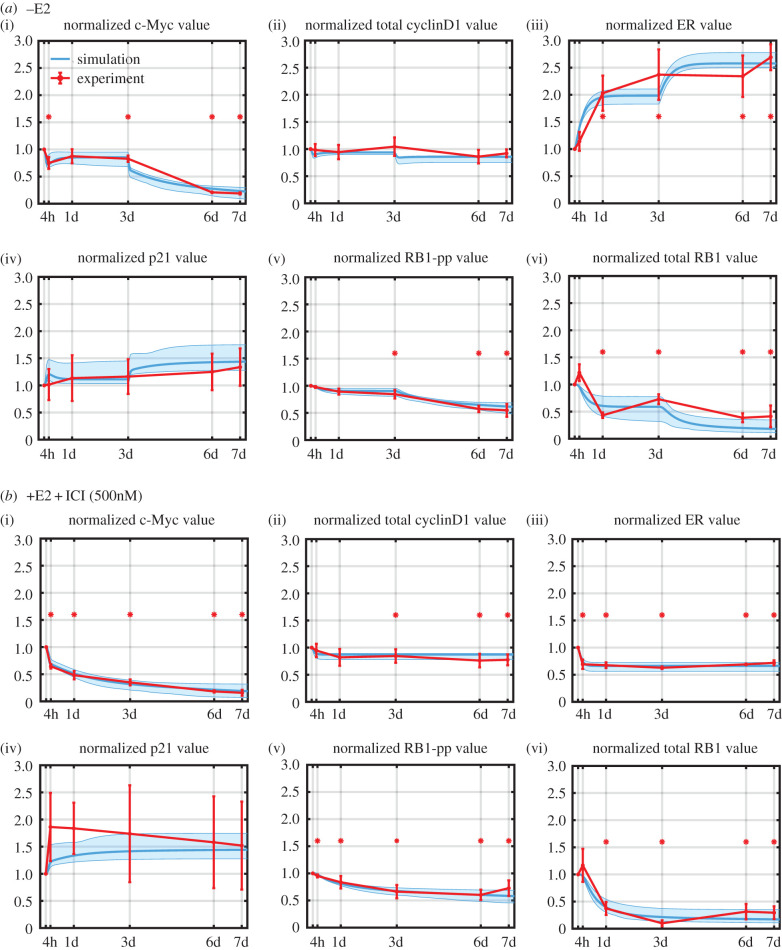
Simulation and experimental results for protein-level changes under the different treatment conditions used for model calibration. The experimental measurements are shown in red (mean value ± s.e., *n* = 3) and the simulation results are shown in cyan (lowest cost simulation as a solid line and the interval containing the central 98% of the cohort simulations as a shaded region). Statistically significant changes from T = 0 are denoted by an asterisk. (*a*) Protein level changes after E2 deprivation over 7 days. (*b*) Protein-level changes after +E2 + ICI treatment over 7 days. ICI concentration is 500 nM and +E2 concentration is 10 nM. The simulation values for total cyclinD1 and total p21 are plotted to enable comparison with experiment.

The training data for estimating the model parameters consisted of time-course measurements of the proteins in red in figure 1*b* for the –E2 and +E2 + ICI treatment conditions. These data are shown in figure 2, and it can be seen that the majority of measurements are statistically significant (asterisks), although the p21 measurements are quite noisy, and there is no real trend in the cyclinD1 data for the –E2 case. However, there are clear trends in most of the data, and it is critical that the model structure is capable of reproducing these trends. Figure 2*a* shows the experimental observations over 7 days (red) in response to –E2. The significant ER increase after treatment can be captured by the model because the half-life of E2:ER (oestrogen-bound ER) is about 3–4 h [30] and the half-life of unbound ER is about 4–5 h [30]. Therefore, the depletion of E2 stabilizes ER and causes the total ER level to increase. The jump in ER after 3 days is due to the medium exchange that decreases the concentration of E2 in the medium that is leached from the cells (see Methods). The small decrease in the cyclinD1 level can be captured by the model since E2:ER is a transcription factor for cyclinD1. By contrast, c-Myc not only decreases at the beginning, but decreases abruptly at 3d and continues to decrease over the remainder of the time course. The model captures the sharp decreases due to changes in the E2 concentration levels at 0d and 3d and the gradual decrease via RB1-pp, which drives the production of c-Myc. As the cells are gradually blocked in G1, RB1-pp decreases steadily, causing a decrease in c-Myc. The noisy p21 data do appear to increase; this is captured in the model as decreasing c-Myc releases its transcriptional inhibition of p21. We should note that there are likely to be many ways in which c-Myc influences RB1-pp, such as activation of cyclinE:Cdk2 kinase activity via Cdc25A [31], but, for simplicity, we have chosen to model c-Myc's effect only through the p21 pathway. The model can capture the reduction in RB1-pp, since this follows from the reductions in c-Myc and cyclinD1.

At first glance, it seems odd that total RB1 should decrease much more than RB1-pp and that this decrease should be associated with the observed decrease in proliferation, as less total RB1 might be expected to create free E2F and promote the G1–S transition. This decrease in total RB1 has been observed by others in response to E2 deprivation [32]. In the model, free RB1 is always much more prevalent than either of the phosphorylated forms. The level of RB1-p is controlled by the cyclinD1 level, since the influence of free RB1 is saturated in the Hill function kinetics and has little effect on the RB1-p level. In turn, the level of RB1-pp is controlled by cyclinE and not influenced significantly by the RB1 level. The model captures the changes in total RB1 through the presumed transcriptional effect of RB1-pp on RB1.

Figure 2*b* shows the experimental observations in response to treatment with ICI. The model can capture the decrease in ER since ICI binds to ER, displacing E2 and enhancing ER degradation via the proteasome [33]. The reduced availability of the transcription factor ER:E2 due to ICI treatment causes changes in c-Myc, cyclinD1, p21, RB1-pp and total RB1 similar to those caused by reduced availability of ER:E2 due to E2 deprivation, and for the same reasons.

### 3.2. Model calibration and the simulation cohort

To calibrate the model, we used a genetic algorithm to search for parameter values minimizing the least-squares cost function of §2.7. To address the fact that the model may not be practically identifiable from the data, we identified 399 other parameter sets that provided a reasonable fit to the data to use as a simulation cohort. Any member of the cohort is a reasonable parameterization of the model and when we use the model for prediction we simulate all 400 parameter sets. The resulting spread in the predictions enables us to see how well the data used to calibrate the model constrain the prediction. The coefficients of variation of the parameters and the variations in the cost function for the simulation cohort are plotted in electronic supplementary material, figure S3.

The final results of calibrating the model to the training data are shown in figure 2. The results are quite reasonable, given the simplicity of our model. The spread of results from the simulation cohort reflects both the variation in the parameters for the chosen cohort as well as the sensitivity of the model to these parameters. The least spread is exhibited by cyclinD1 and RB1-pp. Although RB1-pp is not extremely sensitive to the parameters driving it (see sensitivity analysis in the electronic supplementary material), proliferation is very sensitive to RB1-pp level and forces its level to be heavily constrained. CyclinD1, on the other hand, is simply not very sensitive to the parameters driving it and the limited coefficients of variation of these parameters in the cohort result in a small spread.

Of course, there is always the question of whether the fit is reasonable because the model captures the essentials of the mechanism or simply because enough parameters were added to the model. In the following, we argue that our ability to add a new drug to the model without recalibration, and the ability of the model to predict drug combinations, shows that the model is capturing some of the essential mechanism.

### 3.3. Adding a new drug to the model requires limited new data

**Figure 3.**
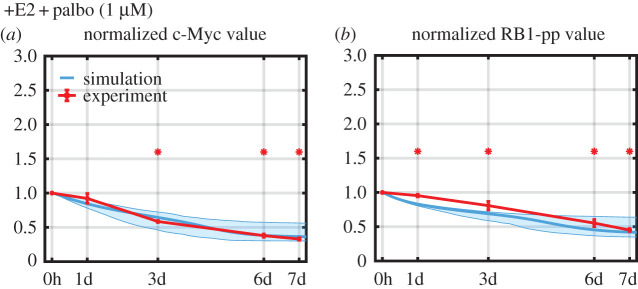
Simulation and experimental results for protein-level changes in response to palbociclib. Simulations are shown in cyan and experimental measurements in red (mean value ± s.e., *n* = 3). Statistically significant changes from *T* = 0 are denoted by asterisks.

If the model is capturing the basic mechanism driving protein changes, adding a new drug to the model should require only calibrating a few new parameters associated with the new drug. Such calibration should not require measuring the response of all proteins in the model to the new drug, but only a few key proteins. We illustrate this by incorporating palbociclib, which has been used clinically in combination with endocrine therapy [5–7,10,34], into our model. Palbociclib inhibits Cdk4/6 kinase activity, reducing RB1 phosphorylation, primarily at S780/S795 [7,35], and ultimately leading to reduced hyperphosphorylation of RB1 and cell cycle arrest. To calibrate the binding and unbinding parameters associated with palbociclib in our model, we measured only two proteins, c-Myc and RB1-pp (S612, associated with proliferation but not directly affected by palbociclib [25]), which should experience strong downstream reactions to the drug. Figure 3 shows the results of calibrating the model to 1 μM of palbociclib added to the +E2 medium. As expected, the inhibition of cyclinD1:Cdk4/6 kinase activity by palbociclib decreases the RB1-pp level. This, in turn, leads to decreased transcription of c-Myc, causing the c-Myc level to decrease.

### 3.4. The proliferation results can be explained by the RB1-pp level

Proliferation is a primary endpoint for treatment decisions, so it is important for the model to be able to capture how proliferation changes with treatment. When considering treatments that produce G1 arrest, the key transition governing the execution of the cell cycle is the transition from the G1 phase to the S phase. A major determinant of the transition is the phosphorylation status of RB1 [36], but there are many other factors affecting the transition (see the discussion in the electronic supplementary material under Biological Justification of the Model).

Based on our experimental data, we assume that the rate of transition, hence of proliferation, in our model of an asynchronous population of cells is governed by the level of RB1-pp according to

$$\frac{\text{dcell}}{dt}=k_{\mathrm{pro}}\times \left(1+k_{\;proRB1pp}\times \frac{RB1pp^{\;p_{\;proRB1pp_{2}}}}{\;p_{\;proRB1pp_{1}}^{\;p_{\;proRB1pp_{2}}}+RB1pp^{\;p_{\;proRB1pp_{2}}}}\right)\times \text{cell}\times \left(1-\frac{\text{cell}}{k_{\mathrm{carrying}}}\right).$$

The rate of cellular proliferation is modelled as proportional to the current number of cells subject to a carrying capacity constraint (logistic growth). The proportionality constant depends on the RB1-pp level via a Hill function. We expect proliferation to be near maximal at the pre-treatment RB1-pp level and, in our experimental data, proliferation essentially stops when the RB1-pp level reaches half of its normalized value, necessitating a relatively large Hill exponent of 6. The carrying capacity, assumed constant across all experiments, is used to account for the fact that in the control condition the cells approach confluence in 7 days; it has little effect on the other, slower growing, conditions.

**Figure 4.**
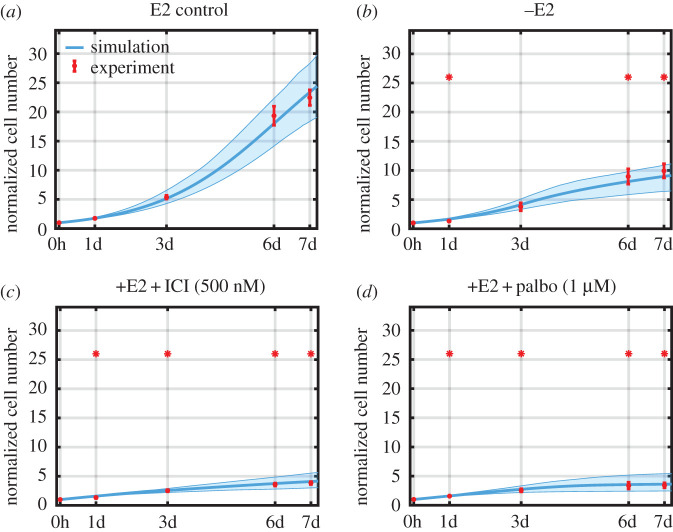
Model simulations and experimental measurements of the normalized cell numbers under different treatment conditions. Experimental counts are in red (mean value ± s.e., *n* = 3) and simulations are in cyan. ICI concentration is 500 nM, palbociclib concentration is 1 µM and E2 concentration is 10 nM. Cell numbers are normalized to *T* = 0 value. Statistically significant changes from the +E2 control are denoted by asterisks.

Figure 4 shows that the model does a good job of matching the experimental proliferation results for the untreated (+E2), deprived (–E2), ICI-treated and palbociclib-treated cases on which the model was calibrated. This helps validate our claim that proliferation in these cases can be modelled using the RB1-pp (S612) level.

### 3.5. The model can predict the effect of combination treatments

**Figure 5.**
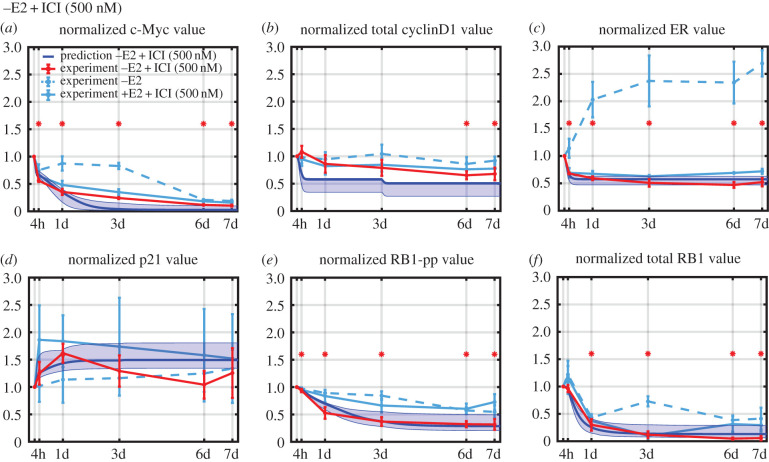
Prediction simulations for protein-level changes in response to combination E2 deprivation +ICI therapy. ICI concentration is 500 nM. Experimental results are in red (mean value ± s.e., *n* = 3), simulations are in blue and monotherapy experimental results are in cyan. Statistically significant changes from *T* = 0 are denoted by asterisks.

To partially validate the model, we test its ability to predict the effect of combination therapies on protein levels and cell proliferation. Figure 5 shows how the model simulations of combining E2 deprivation and ICI compare to the experimental results. With the exception of cyclinD1, the predictions match the experimental results for the six measured protein species well. By reducing the supply of E2 and the ER level simultaneously, larger changes in the protein levels are observed than with either monotherapy, as would be expected.

**Figure 6.**
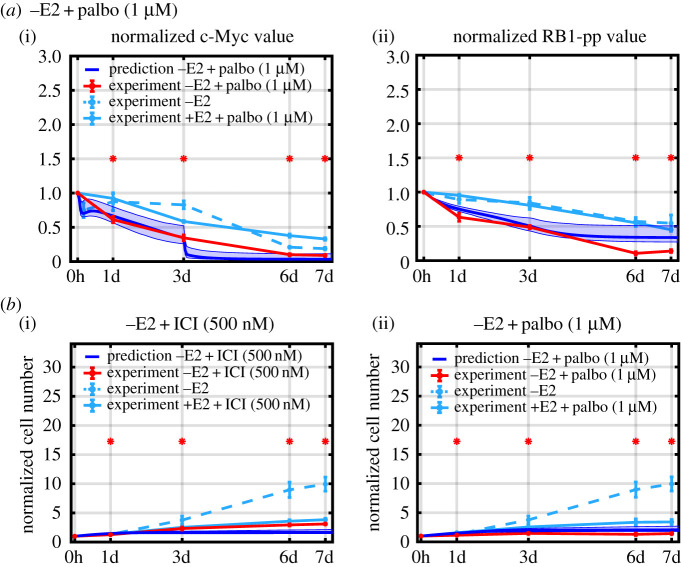
Comparison of model predictions with experiment for combination therapies. Experimental data (red), simulations (blue) and monotherapy experimental results (cyan). (*a*) Prediction of c-Myc and RB1-pp levels in response to –E2 + palbociclib (1 μM) combination treatment. (*b*) Prediction of proliferation in response to –E2 + ICI and –E2 + palbociclib (1 μM) combination treatments. Asterisks denote statistically significant changes from *T* = 0 for proteins, and from the +E2 control for proliferation.

The combination of E2 deprivation and palbociclib attacks a key G1 kinase, cyclinD1:Cdk4/6, by both reducing the level of cyclinD1 and inactivating Cdk4/6. As expected, the combination reduces the level of c-Myc and RB1-pp to a greater extent than either monotherapy. The predictions of the model are compared with experimental protein measurements in figure 6*a*. The agreement between measured and predicted c-Myc is good, but the simulation misses the low level of RB1-pp at the final two time points. Since proliferation in the model essentially stops when the normalized RB1-pp level falls below 0.5, it is still possible to capture the proliferation well while somewhat overestimating the RB1-pp level.

Figure 6*b* shows that the model also predicts the decreased proliferation in response to the combination therapies quite well. This follows from the fact that RB1-pp decreases more in response to the combinations than to either monotherapy.

### 3.6. Local sensitivity analysis of protein levels and proliferation

**Figure 7.**
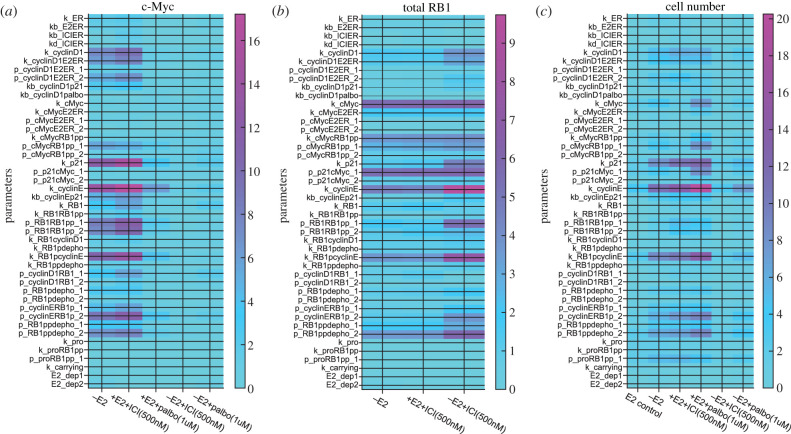
Local sensitivity analysis of the effect on c-Myc level, total RB1 level and cell number of each model parameter. Each of the parameters is changed by ±5% and the sensitivity at day 7 for each treatment is plotted.

A local sensitivity analysis is used to check if the output of our model is very sensitive to certain parameters. The sensitivity coefficient for a given model output and a given parameter is the per cent change in the model output divided by the per cent change in the parameter value. To calculate this coefficient, we changed the parameter by ±5% from its best-fit value, so the sensitivity coefficient is not unduly local. In figure 7, we plot the sensitivity coefficients for the most sensitive outputs in our model, c-Myc, total RB1 and cell number, to each model parameter at the day 7 time point. The sensitivity coefficients of the other proteins are shown in the electronic supplementary material and are of the order of 4 or less, indicating relatively low sensitivity.

The most significant sensitivity for c-Myc occurs for the ICI treatment case and involves parameters related to the basal translation of cyclinE and p21 and the phosphorylation of RB1-p. This is not surprising as these parameters regulate the level of RB1-pp, which helps drive the c-Myc level in our model. Total RB1 is most sensitive for the –E2 + ICI case, and the parameters producing the greatest sensitivity involve basal transcription of cyclinE and c-Myc, the downregulation of p21 by c-Myc, phosphorylation of RB1-p by cyclinE and the dephosphorylation of RB1-pp. These parameters all converge on the level of RB1-pp.

Cell number is most sensitive in the ICI and palbociclib treatment cases and its greatest sensitivity is to parameters involving basal transcription of cyclinE and its phosphorylation of RB1-p. Again, these parameters drive the level of RB1-pp, which in turn drives proliferation. The fact that cell proliferation is essentially modelled as exponential accounts for the large sensitivity.

### 3.7. The model can be used to explore the effect of sequential therapies

While the initial response of patients to a mono- or combination therapy is often promising, in many cases, resistance to continuous therapy eventually arises (e.g. palbociclib [10,11], letrozole [8,12] and ICI [37]). The resistance mechanisms are varied, and in many cases not well understood, but can include protein overexpression, epigenetic changes, gene mutation and gene amplification or deletion [8–12,38]. Our idea to address some of these mechanisms is straightforward: alternate various therapies, perhaps including drug holidays, in a fixed cycle to significantly suppress proliferation of tumour cells, but not target one particular mechanism non-stop to avoid selecting for resistance mechanisms. The hope is that such an approach can hold off, or at least delay, the onset of resistance.

Optimizing sequential therapies under various restrictions is likely to be impractical experimentally because of the large number of variations in dosing and timing of multiple therapies that must be considered. On the other hand, a reliable mathematical model may be able to sort through the huge number of possibilities to find particularly promising therapy protocols that can be tested experimentally.

**Figure 8.**
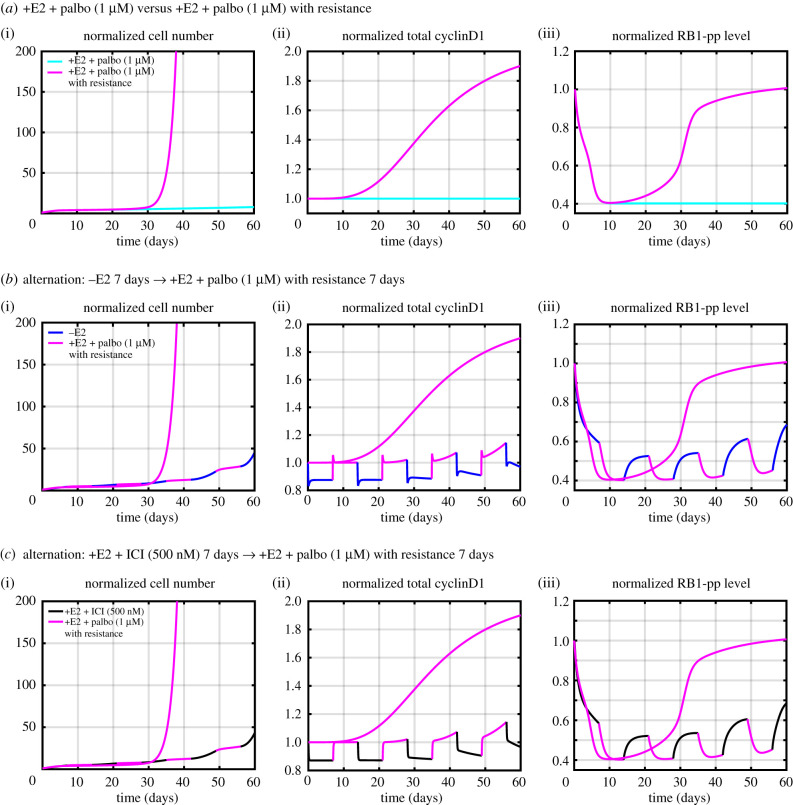
Model simulations of possible alternating therapies compared with a continuous monotherapy with an added resistance mechanism. (*a*) Continuous palbociclib therapy with and without the resistance mechanism are compared. (*b*) Continuous palbociclib therapy is compared with alternation of E2 deprivation and palbociclib therapies. (*c*) Continuous palbociclib therapy is compared with alternation of ICI and palbociclib therapies. Doses: E2 (10 nM), ICI (500 nM), palbociclib (1 μM). The simulations use the lowest cost parameter set, and all simulations in (*b*) and (*c*) include the resistance mechanism.

To illustrate the basic idea with our current model, we consider resistance to palbociclib, which is frequently associated with the overexpression of Cdk6, which increases over time with continuous palbociclib therapy [39,40]. Presumably, the increasing Cdk6 eventually binds enough palbociclib to free up cyclinD1:Cdk4/6 to phosphorylate RB1 and move the cell toward proliferation. In our model, we did not include Cdk4/6 explicitly but rather assumed that all free cyclinD1 was complexed with Cdk4/6 and, therefore, active. Palbociclib inhibits this activity in the model by binding to cyclinD1 and holding it inactive. We mimic the resistance effect of increased Cdk6 sequestering palbociclib by slowly increasing the level of cyclinD1 transcription under palbociclib therapy and allowing it to slowly decrease when palbociclib is withdrawn. So palbociclib is sequestered by cyclinD1, rather than Cdk6, in the model. Modelling details are in the electronic supplementary material, equations 40 and 41. Figure 8*a* shows how adding this resistance mechanism changes the predicted results of monotherapy, with resistance emerging after 30 days.

Figure 8*b* compares a continuous therapy of palbociclib with a therapy that alternates E2 deprivation with palbociclib for one-week periods in a repeating cycle. While the cyclinD1 and RB1-pp levels slowly increase under the alternating therapy, the onset of resistance is effectively delayed. Figure 8*c* shows a similar effect when treatment with ICI and palbociclib are alternated with one-week periods.

## 4. Discussion

In this study, we created a relatively simple mathematical model to capture the effects of ER signalling on key cell cycle proteins governing progression through the G1–S checkpoint in breast cancer cells. The model was calibrated using experimental monotherapy data and was capable of predicting the experimental data from combination therapies. We further showed that it was relatively easy to add in the effect of a new drug that impinged on this checkpoint without having to measure the response of all the proteins in the model to the new drug or to recalibrate the previously determined parameters of the model. Finally, we linked the level of hyperphosphorylated RB1 (S612) to the proliferation rate and showed that this linkage could capture the proliferation observed in our experimental data.

Clearly, our model is a highly simplified version of the actual system. We assume that the behaviour of a population of cells can be adequately modelled by allowing the average levels of species in the population to interact according to a simplified version of the mechanism existing in individual cells. While there are theoretical objections to such a model (the interaction rate between A and B in a population is not necessarily proportional to the average concentration of A multiplied by the average concentration of B), this approach has produced useful models in other contexts [13]. One consequence of this approach is that our model, unlike the model of a single cell, will not exhibit a bistable switch governing the G1–S transition [17], as a population will not completely stop cycling even though individual cells will. Probably, the fact we are trying to model the behaviour of a large population of asynchronous cells allows the model to be simpler than if we were trying to model the intricacies of an individual cell.

Our ultimate goal is to use the model to derive alternating therapies that may stave off the development of resistance often associated with continuously applied therapies [41,42]. We illustrated some possibilities along this line, but clearly additional experimental confirmation is required. Confirmation that the model can accurately predict proliferation over longer time scales and in response to switching therapies will be the subject of future work, as will confirmation that cells subjected to alternating therapies are truly less resistant than cells subjected to continuous therapy.

We should also note that the treatments we are currently considering all impinge on the G1–S transition. Thus, a resistance mechanism that completely eradicates the G1–S checkpoint, such as loss of RB1, is likely to be unaffected by our approach. Drugs that arrest the cell cycle at a different point, for example G2, or that target pathways resulting in cell death will be necessary to address such cases. Drugs that induce significant cell death will also be necessary if we want to provide a drug holiday. While the holiday may help prevent the development of resistance, it will also allow significant proliferation and holding down the net proliferation will require a drug that can kill cells [43]. Finally, we realize that successful results *in vitro* are potentially a long way off from success in animal models or human tumours, but it is the place to start for mechanism-based modelling.

## Supplementary Material

Supporting Information: Details of the Mathematical Model

## Supplementary Material

Matlab Code File 1: Main File

## Supplementary Material

Matlab Code File 2: Differential Equations

## Supplementary Material

Matlab Code File 3: Helper Function

## Supplementary Material

SBML Version of the Model

## Supplementary Material

Raw Data from the Experiments: Western Blot Intensities and Coulter Counter Counts

## Supplementary Material

Parameter Cohort: Listing of the 400 Parameter Sets in the Cohort

## Data accessibility

All relevant data and code are included in the manuscript and the electronic supplementary material. This article has no additional data.

## References

[1] Siegel RL, Miller KD, Jemal A 2019 Cancer statistics. CA Cancer J. Clin. 69, 7–34. (10.3322/caac.21551) 30620402

[2] Mandlekar S, Kong AN 2001 Mechanisms of tamoxifen-induced apoptosis. Apoptosis 6, 469–477. (10.1023/A:1012437607881) 11595837

[3] Riggins RB, Bouton AH, Liu MC, Clarke R 2005 Antiestrogens, aromatase inhibitors, and apoptosis in breast cancer. Vitam. Horm. 71, 201–237. (10.1016/S0083-6729(05)71007-4) 16112269

[4] Fanning SW, Greene GL 2019 Next-generation ERalpha inhibitors for endocrine-resistant ER+ breast cancer. Endocrinology 160, 759–769. (10.1210/en.2018-01095) 30753408

[5] Finn RS et al. 2015 The cyclin-dependent kinase 4/6 inhibitor palbociclib in combination with letrozole versus letrozole alone as first-line treatment of oestrogen receptor-positive, HER2-negative, advanced breast cancer (PALOMA-1/TRIO-18): a randomised phase 2 study. Lancet Oncol. 16, 25–35. (10.1016/S1470-2045(14)71159-3) 25524798

[6] Cristofanilli M et al. 2016 Fulvestrant plus palbociclib versus fulvestrant plus placebo for treatment of hormone-receptor-positive, HER2-negative metastatic breast cancer that progressed on previous endocrine therapy (PALOMA-3): final analysis of the multicentre, double-blind, phase 3 randomised controlled trial. Lancet Oncol. 17, 425–439. (10.1016/S1470-2045(15)00613-0) 26947331

[7] Stearns V, Brufsky AM, Verma S, Cotter MJ, Lu DR, Dequen F, Joy AA 2018 Expanded-access study of palbociclib in combination with letrozole for treatment of postmenopausal women with hormone receptor-positive, HER2-negative advanced breast cancer. Clin. Breast Cancer 18, e1239–e1245. (10.1016/j.clbc.2018.07.007) 30172722

[8] Ma CX, Reinert T, Chmielewska I, Ellis MJ 2015 Mechanisms of aromatase inhibitor resistance. Nat. Rev. Cancer. 15, 261–275. (10.1038/nrc3920) 25907219

[9] Musgrove EA, Sutherland RL 2009 Biological determinants of endocrine resistance in breast cancer. Nat. Rev. Cancer 9, 631–643. (10.1038/nrc2713) 19701242

[10] Herrera-Abreu MT et al. 2016 Early adaptation and acquired resistance to CDK4/6 inhibition in estrogen receptor-positive breast cancer. Cancer Res. 76, 2301–2313. (10.1158/0008-5472.CAN-15-0728) 27020857 PMC5426059

[11] Gu G, Dustin D, Fuqua SAW 2016 Targeted therapy for breast cancer and molecular mechanisms of resistance to treatment. Curr. Opin Pharmacol. 31, 97–103. (10.1016/j.coph.2016.11.005) 27883943

[12] Tilghman SL et al. 2013 Proteomic signatures of acquired letrozole resistance in breast cancer: suppressed estrogen signaling and increased cell motility and invasiveness. Mol. Cell Proteomics 12, 244–2455. (10.1074/mcp.M112.023861) PMC3769322 23704778

[13] Kirouac DC et al. 2013 Computational modeling of ERBB2-amplified breast cancer identifies combined ErbB2/3 blockade as superior to the combination of MEK and AKT inhibitors. Sci. Signal. 6, ra68 (10.1126/scisignal.2004008) 23943608

[14] Simms K, Bean N, Koerber A 2012 A mathematical model of cell cycle progression applied to the MCF-7 breast cancer cell line. Bull. Math. Biol. 74, 736–767. (10.1007/s11538-011-9700-2) 22083513

[15] Ibrahim F, Huang B, Xing JZ, Roa W, Gabos S 2010 A mathematical model of *in vitro* estrogen-related cancer cell growth based on cell-cycle mechanism. IFAC Proc. Vol. (IFAC-PapersOnline) 43, 263–268. (10.3182/20100707-3-BE-2012.0039)

[16] Chen C, Baumann WT, Clarke R, Tyson JJ 2013 Modeling the estrogen receptor to growth factor receptor signaling switch in human breast cancer cells. FEBS Lett. 587, 3327–3334. (10.1016/j.febslet.2013.08.022) 23994522 PMC3893882

[17] Yao G, Tan C, West M, Nevins JR, You L 2011 Origin of bistability underlying mammalian cell cycle entry. Mol. Syst. Biol. 7, 1–10. (10.1038/msb.2011.19) PMC3101952 21525871

[18] Yao G, Lee TJ, Mori S, Nevins JR, You L 2008 A bistable Rb-E2F switch underlies the restriction point. Nat. Cell Biol. 10, 476–482. (10.1038/ncb1711) 18364697

[19] Novák B, Tyson JJ 2004 A model for restriction point control of the mammalian cell cycle. J. Theor. Biol. 230, 563–579. (10.1016/j.jtbi.2004.04.039) 15363676

[20] Singhania R, Sramkoski RM, Jacobberger JW, Tyson JJ 2011 A hybrid model of mammalian cell cycle regulation. PLoS Comput. Biol. 7, e1001077 (10.1371/journal.pcbi.1001077) 21347318 PMC3037389

[21] Goldbeter A, Ge C 2009 Temporal self-organization of the cyclin/Cdk network driving the mammalian cell cycle. Proc. Natl Acad. Sci. USA 106, 21 643–21 648. (10.1073/pnas.0903827106) PMC2799800 20007375

[22] Shajahan AN, Dobbin ZC, Hickman FE, Dakshanamurthy S, Clarke R 2012 Tyrosine-phosphorylated caveolin-1 (Tyr-14) increases sensitivity to paclitaxel by inhibiting BCL2 and BCLxL proteins via c-Jun N-terminal kinase (JNK). J. Biol. Chem. 287, 17 682–17 692. (10.1074/jbc.M111.304022) PMC3366801 22433870

[23] Shajahan AN, Wang A, Decker M, Minshall RD, Liu MC, Clarke R 2007 Caveolin-1 tyrosine phosphorylation enhances paclitaxel-mediated cytotoxicity. J. Biol. Chem. 282, 5934–5943. (10.1074/jbc.M608857200) 17190831

[24] MacDonald JI, Dick FA 2012 Posttranslational modifications of the retinoblastoma tumor suppressor protein as determinants of function. Genes Cancer 3, 619–633. (10.1177/1947601912473305) 23634251 PMC3636757

[25] Lents NH, Gorges LL, Baldassare JJ 2006 Reverse mutational analysis reveals threonine-373 as a potentially sufficient phosphorylation site for inactivation of the retinoblastoma tumor suppressor protein (pRB). Cell Cycle 5, 1699–1707. (10.4161/cc.5.15.3126) 16880741

[26] Strobl JS, Lippman ME 1979 Prolonged retention of estradiol by human breast cancer cells in tissue culture. Cancer Res. 39, 3319 LP–333327. 476661

[27] Masamura S, Santner SJ, Heitjan DF, Santen RJ 1995 Estrogen deprivation causes estradiol hypersensitivity in human breast cancer cells. J. Clin. Endocrinol. Metab. 80, 2918–2925. (10.1210/jcem.80.10.7559875) 7559875

[28] Mathworks. 2014 MATLAB and Global Optimization Toolbox, release 2014a. The MathWorks, Inc., Natick, MA.

[29] Jove M et al. 2019 Cellular uptake and efflux of palbociclib *in vitro* in single cell and spheroid models. J. Pharmacol. Exp. Ther. 370, 242–251. (10.1124/jpet.119.256693) 31189729

[30] Wijayaratne AL, McDonnell DP 2001 The human estrogen receptor-α is a ubiquitinated protein whose stability is affected differentially by agonists, antagonists, and selective estrogen receptor modulators. J. Biol. Chem. 276, 35 684–35 692. (10.1074/jbc.M101097200) 11473106

[31] Galaktionov K, Chen X, Beach D 1996 Cdc25 cell-cycle phosphatase as a target of c-myc. Nature **382**, 511–517. (10.1038/382511a0) 8700224

[32] Carroll JS, Prall OWJ, Musgrove EA, Sutherland RL 2000 A pure estrogen antagonist inhibits cyclin E-Cdk2 activity in MCF-7 breast cancer cells and induces accumulation of p130-E2F4 complexes characteristic of quiescence. J. Biol. Chem. 275, 38 221–38 229. (10.1074/jbc.M004424200) 10991938

[33] Wittmann BM, Sherk A, McDonnell DP 2007 Definition of functionally important mechanistic differences among selective estrogen receptor down-regulators. Cancer Res. 67, 9549–9560. (10.1158/0008-5472.CAN-07-1590) 17909066

[34] Finn RS et al. 2014 Abstract CT101: Final results of a randomized phase II study of PD 0332991, a cyclin-dependent kinase (CDK)-4/6 inhibitor, in combination with letrozole vs letrozole alone for first-line treatment of ER+/HER2- advanced breast cancer (PALOMA-1; TRIO-18). Cancer Res. 74, CT101 (10.1158/1538-7445.AM2014-CT101) PMC7383036 32683565

[35] Fry DW et al. 2004 Specific inhibition of cyclin-dependent kinase 4/6 by PD 0332991 and associated antitumor activity in human tumor xenografts. Mol. Cancer Ther. 3, 1427–1438. 15542782

[36] Giacinti C, Giordano A 2006 RB and cell cycle progression. Oncogene 25, 5220–5227. (10.1038/sj.onc.1209615)5(5):). 467–474. 16936740

[37] McClelland RA et al. 2001 Enhanced epidermal growth factor receptor signaling in MCF7 breast cancer cells after long-term culture in the presence of the pure antiestrogen ICI 182,780 (Faslodex). Endocrinology 142, 2776–2788. (10.1210/endo.142.7.8259) 11415996

[38] Butt AJ, McNeil CM, Musgrove EA, Sutherland RL 2005 Downstream targets of growth factor and oestrogen signalling and endocrine resistance: the potential roles of c-Myc, cyclin D1 and cyclin E. Endocr Relat. Cancer. 12(Suppl. 1), 47–60. (10.1677/erc.1.00993) 16113099

[39] Yang C et al. 2017 Acquired CDK6 amplification promotes breast cancer resistance to CDK4/6 inhibitors and loss of ER signaling and dependence. Oncogene 36, 2255–2264. (10.1038/onc.2016.379) 27748766 PMC5393973

[40] Cornell L, Wander SA, Visal T, Wagle N, Shapiro GI 2019 MicroRNA-mediated suppression of the TGF-beta pathway confers transmissible and reversible CDK4/6 inhibitor resistance. Cell Rep. 26, 2667–2680. (10.1016/j.celrep.2019.02.023) 30840889 PMC6449498

[41] Smith MP et al. 2016 Inhibiting drivers of non-mutational drug tolerance is a salvage strategy for targeted melanoma therapy. Cancer Cell. 29, 270–284. (10.1016/j.ccell.2016.02.003.) 26977879 PMC4796027

[42] Sharma SV et al. 2010 A chromatin-mediated reversible drug-tolerant state in cancer cell subpopulations. Cell 141, 69–80. (10.1016/j.cell.2010.02.027) 20371346 PMC2851638

[43] Das Thakur M, Salangsang F, Landman AS, Sellers WR, Pryer NK, Levesque MP, Dummer R, McMahon M, Stuart DD 2013 Modelling vemurafenib resistance in melanoma reveals a strategy to forestall drug resistance. Nature 494, 251 (10.1038/nature11814) 23302800 PMC3930354

