# Supplementary material for: Mathematical modelling of breast cancer cells in response to endocrine therapy and Cdk4/6 inhibition
Source: J R Soc Interface. 2020 Aug 26;17(169):20200339. doi: 10.1098/rsif.2020.0339 (PMC7482571; doi:10.1098/rsif.2020.0339)
Supplement: Supporting Information: Details of the Mathematical Model [file rsif20200339supp1.pdf]

Supporting information

Mathematical modeling of breast cancer cells in response to endocrine therapy and Cdk4/6 inhibition

Journal of the Royal Society Interface

Wei He, Diane M. Demas, Isabel P. Conde, Ayesha N. Shajahan-Haq, William T. Baumann

Biological Justification for the Model

Endocrine therapy, targeting ER signaling, is widely-used to treat ER+ breast cancer patients [1-7]. Two common endocrine therapies are E2 deprivation, via aromatase inhibitors (AIs) [5, 8], and ER down-regulators such as ICI182,780 (ICI; fulvestrant, Faslodex) [7, 9, 10]. AIs, like letrozole or anastrozole, are used in postmenopausal women for the treatment of breast cancer [8]. AIs lower the E2 level by inhibiting aromatase, which is an enzyme responsible for a key step in the synthesis of E2 [5]. The steroidal anti-estrogen ICI is also used in postmenopausal women. It competitively blocks E2 binding to the ER and causes a proteasome-dependent degradation of the receptor [11].

The central effect of steroid hormone E2 is growth maintenance and reproduction [12, 13] through the regulation of various processes, including cell proliferation and survival [14]. E2 exerts its effects by binding to nuclear receptors ERα and ERβ [15]. ERα is frequently overexpressed in breast cancer cells and is the major oncogene responsible for E2-induced enhancement of cell proliferation [14]. In the classical signaling mechanism, E2 binds to ERα (E2:ER) in the cytoplasm, producing conformational changes that result in homodimerization, translocation to the nucleus, and binding to estrogen response elements (ERE) in the promoter region of estrogen-responsive genes to regulate transcription [13, 14]. One important class of estrogen-responsive genes regulate progression through the G1 phase of the cell cycle [14, 16]. This progression mainly depends on transcriptional expression of regulatory proteins c-Myc and cyclinD1 [16].

c-Myc responds to E2 stimulation within 15 minutes, making it one of the earliest estrogen-responding genes [17-19]. The rapid induction of c-Myc by E2:ER is via the P2 promoter region of the c-Myc gene, which contains an atypical ERE region [17, 20]. c-Myc is a nuclear transcription factor with high-affinity for DNA-binding and its level is highly correlated with breast cancer cell proliferation [21, 22]. The c-Myc oncogene is usually overexpressed in human breast cancer cells and is an essential positive regulator of the G1-S transition [22]. c-Myc represses transcription of the cyclin-dependent kinase inhibitor $p21^{CIP1/WAF1}$ (p21) by interacting with Miz-1 and several other proteins at the transcriptional start site [23] and transforming Miz-1 from an activator to a repressor [24, 25]. In addition, c-Myc also can transcriptionally repress $p27^{KIP1}$ (p27) expression in breast cancer cells [23] by binding to the initiator element at the start site of the TATA-less p27 promoter [26]. Moreover, the box II domain of c-Myc can interact with the N-terminal DNA-binding region of FOXO3a at the proximal Forkhead element on the p27 promoter. This interaction inhibits the FOXO3a-mediated activation of the p27 promoter [27].

Besides c-Myc, E2:ER also transcriptionally up-regulates cyclinD1, whose expression is key to G1-S progression [28, 29]. ERα binds upstream (-96 and -29bp) of the cyclinD1 promoter region encompassing a cAMP response element (CRE-D1) [28, 30]. The up-regulation is E2-dependent and the AF-1, AF-2 and DNA binding domains of ERα are required [28, 30]. In addition, c-Myc has been reported to transcriptionally up-regulate [31], down-regulate [32-34], or have no effect on cyclinD1 expression level [35-37]. When tested on breast cancer cells [16], induction of c-Myc failed to increase cyclinD1 expression and induction of cyclinD1 had no effect on c-Myc expression in the interval from 3 to 24 hours after induction [16]. Therefore, we model the increased transcription of cyclinD1 and c-Myc as a direct effect of E2:ER.

CyclinD1 binds with cyclin-dependent kinases Cdk4 or Cdk6 (mostly Cdk4 in MCF-7 cells [38, 39]) to form holoenzymes and activate their kinase activity [40]. CyclinD1:Cdk4/6 phosphorylates

retinoblastoma protein (RB1) to a hypophosphorylated form (RB1-p) [16, 41-56]. The Cdk inhibitors p21 and p27, which can bind to the cyclinD1:Cdk4/6 complexes and inhibit their kinase activity [41, 42, 57, 58], are downregulated by c-Myc to further activate the holoenzyme. Since p21 and p27 also inhibit the kinase activity of cyclinE:Cdk2, the increased sequestration of these inhibitors by cyclinD and their downregulation by c-Myc, serve to activate the cyclinE:Cdk2 complex [43, 44, 59]. This mechanism of cyclinE:Cdk2 activation by estrogen treatment is confirmed by several studies [45-47]. Finally, active cyclinE:Cdk2 hyperphosphorylates and fully inactivates RB1 [16, 23, 41, 44, 46, 48, 49, 53-56], which is a key step in the G1-S transition [16, 17, 21, 23, 43-45]. The hyperphosphorylated RB1 (RB1-pp) releases E2F transcription factors that transactivate the genes required for DNA synthesis [23, 43-45]. All of these mechanisms have been well-established and recognized in ER+ breast cancer cells [16-20, 43-48, 59, 60].

There are numerous feedback loops involving E2F in the G1-S transition machinery [61-63]. While it is enticing to include all the well-known mechanisms around the G1-S transition, the limited data we are able to obtain does not warrant the increase in parameters, which would be practically, if not theoretically, unidentifiable.

With regard to proliferation, in the simple situation where total RB1 and total E2F are constant, the level of hyperphosphorylated RB1, RB1-pp, should be related to the proliferation rate. But our experimental data, as well as that of others, shows that total RB1 is not constant in response to endocrine therapy. In fact, the interactions between the RB1 and E2F families of proteins is quite complicated: there are three types of pocket proteins that bind E2F (RB1, p107, and p130) and nine types of E2F (E2F1-9), not to mention the 15 phosphorylation sites on RB1. In addition, p130 is known to increase in response to endocrine therapy. From our experimental data it appears that proliferation closely follows the level of RB1-pp (phosphorylation on site S612) and so in our simplified model we use the RB1-pp level to drive changes in proliferation.

The two common endocrine therapies used in this study, E2 deprivation and ER down-regulation, decrease the transcription factor E2:ER, down-regulate c-Myc and cyclinD1, increase p21 and p27, and inhibit cyclinD1:Cdk4/6 and cyclinE:Cdk2 kinase activity [49-53, 64]. The consequence of this decreased kinase activity is reduced phosphorylation of RB1, which limits the transcriptional activity of E2F, impeding the G1-S transition and arresting the cell in a state with characteristics of quiescence [38, 49, 50, 51, 53, 64, 65].

Model

Table S1. Model variable description

Variable name	Description	Initial value	Half-life
(1) *ER*	Estrogen receptor	0.002μM	4-5h [66]
(2) *E2ER*	Estrogen bound estrogen receptor	0.068μM	3-4h [66]
(3) *ICIER*	ICI 182,780 bound estrogen receptor	0μM	< 3-4h [66]
(4) *cyclinD1*	Protein cyclinD1	0.183μM	0.4h [67]
(5) *cyclinD1p21*	p21 bound cyclinD1 protein	0.219μM	-
(6) *cyclinD1palbo*	palbociclib bound cyclinD1 protein	0μM	-
(7) *cMyc*	Protein c-Myc	6.535μM	0.333h [68]
(8) *p21*	Protein p21	0.102μM	0.33-1h [69]
(9) *cyclinE*	Protein cyclinE	0.306μM	0.5h [70]
(10) *cyclinEp21*	p21 bound cyclinE protein	0.274μM	-
(11) *RB1*	Retinoblastoma protein	76.857μM	2-3h [71]
(12) *RB1p*	Hypophosphorylated RB1 (RB1-p)	0.481μM	2-3h [71]

(13) *RB1pp*	Hyperphosphorylated RB1 (RB1-pp)	7.306μM	>4h [71]
(14) *cell*	Cell number	1a.u.	-

a.u. arbitrary units

Table S2. Model parameter description

Parameter name	Description	Value	Fixed or calibrated
(1) k_{ER}	Translation rate of ERα	0.02μM/hour	Calibrated
(2) kd_{ER}	Degradation rate of ERα	0.10/hour	Fixed
(3) kd_{E2ER}	Degradation rate of E2ER	0.30/hour	Fixed
(4) kb_{E2ER}	Binding rate between E2 and ERα	4266.27/(hour×μM)	Calibrated
(5) kub_{E2ER}	Unbinding rate between E2 and ERα	1.0/hour	Fixed
(6) kb_{ICIER}	Binding rate between ICI and ERα	206.80/(hour×μM)	Calibrated
(7) kub_{ICIER}	Unbinding rate between ICI and ERα	1.0/hour	Fixed
(8) kd_{ICIER}	Degradation rate of ICIER	0.52/hour	Calibrated
(9) $k_{cyclinD1}$	Translation rate of cyclinD1	0.14μM/hour	Calibrated
(10) $kd_{cyclinD1}$	Degradation rate of cyclinD1	1.73/hour	Fixed
(11) $k_{cyclinD1E2ER}$	Increased cyclinD1 translation by E2ER	11.57	Calibrated
(12) $p_{cyclinD1E2ER_1}$	Parameter 1 of cyclinD1 increased translation by E2ER	2.61μM	Calibrated
(13) $p_{cyclinD1E2ER_2}$	Parameter 2 of cyclinD1 increased translation by E2ER	0.17	Calibrated

(14) $kb_{cyclinD1p21}$	Binding rate between cyclinD1 and p21	32.09/(hour×μM)	Calibrated
(15) $kub_{cyclinD1p21}$	Unbinding rate between cyclinD1 and p21	1/hour	Fixed
(16) $kb_{cyclinD1palbo}$	Binding rate between cyclinD1 and palbociclib	5.18/(hour×μM)	Calibrated
(17) $kub_{cyclinD1palbo}$	Unbinding rate between cyclinD1 and palbociclib	1/hour	Fixed
(18) k_{cMyc}	Translation rate of c-Myc	0.33μM/hour	Calibrated
(19) kd_{cMyc}	Degradation rate of c-Myc	2.31/hour	Fixed
(20) $k_{cMycE2ER}$	Increased translation of c-Myc by E2ER	14.18	Calibrated
(21) $p_{cMycE2ER_1}$	Parameter 1 of c-Myc increased translation by E2ER	0.03μM	Calibrated
(22) $p_{cMycE2ER_2}$	Parameter 2 of c-Myc increased translation by E2ER	6.99	Calibrated
(23) $k_{cMycRB1pp}$	Increased translation of c-Myc by RB1-pp	37.30	Calibrated
(24) $p_{cMycRB1pp_1}$	Parameter 1 of c-Myc increased translation by RB1-pp	5.50μM	Calibrated
(25) $p_{cMycRB1pp_2}$	Parameter 2 of c-Myc increased translation by RB1-pp	5.36	Calibrated
(26) k_{p21}	Translation rate of p21	1.29μM/hour	Calibrated
(27) kd_{p21}	Degradation rate of p21	1.39/hour	Fixed
(28) $p_{p21cMyc_1}$	Parameter 1 of p21 inhibited translation by c-Myc	9.24μM	Calibrated
(29) $p_{p21cMyc_2}$	Parameter 2 of p21 inhibited translation by c-Myc	2.44	Calibrated
(30) $k_{cyclinE}$	Translation rate of cyclinE	0.81μM/hour	Calibrated
(31) $kd_{cyclinE}$	Degradation rate of cyclinE	1.39/hour	Fixed
(32) $kb_{cyclinEp21}$	Binding rate between cyclinE and p21	20.94/(hour×μM)	Calibrated

(33) $kub_{cyclinEp21}$	Unbinding rate between cyclinE and p21	1.0/hour	Fixed
(34) k_{RB1}	Translation rate of RB1	3.27μM/hour	Calibrated
(35) kd_{RB1}	Degradation rate of RB1	0.35/hour	Fixed
(36) $k_{RB1RB1pp}$	Increased RB1 translation by RB1-pp	390.87μM/hour	Calibrated
(37) $p_{RB1RB1pp_1}$	Parameter 1 of RB1 increased translation by RB1-pp	10.76μM	Calibrated
(38) $p_{RB1RB1pp_2}$	Parameter 2 of RB1 increased translation by RB1-pp	7	Calibrated
(39) $k_{RB1cyclinD1}$	Phosphorylation rate of RB1 by cyclinD1	15.01/hour	Calibrated
(40) $k_{RB1pdepho}$	Dephosphorylation rate of RB1-p	23.62μM/hour	Calibrated
(41) kd_{RB1p}	Degradation rate of RB1-p	0.80/hour	Fixed
(42) $k_{RB1pcyclinE}$	Phosphorylation rate of RB1-p by cyclinE	5.37/hour	Calibrated
(43) $k_{RB1ppdepho}$	Dephosphorylation rate of RB1-pp	9.27μM/hour	Calibrated
(44) kd_{RB1pp}	Degradation rate of RB1-pp	0.05/hour	Fixed
(45) $p_{cyclinD1RB1_1}$	Parameter 1 of RB1 phosphorylation by cyclinD1	14.01μM	Calibrated
(46) $p_{cyclinD1RB1_2}$	Parameter 2 of RB1 phosphorylation by cyclinD1	3.19	Calibrated
(47) $p_{RB1pdepho_1}$	Parameter 1 of RB1-p dephosphorylation	0.68μM	Calibrated
(48) $p_{RB1pdepho_2}$	Parameter 2 of RB1-p dephosphorylation	7	Calibrated
(49) $p_{cyclinERB1p_1}$	Parameter 1 of RB1-p phosphorylation by cyclinE	6.35μM	Calibrated
(50) $p_{cyclinERB1p_2}$	Parameter 2 of RB1-p phosphorylation by cyclinE	0.35	Calibrated
(51) $p_{RB1ppdepho_1}$	Parameter 1 of RB1-pp dephosphorylation	59.85μM	Calibrated

(52) $p_{RB1ppdepho_2}$	Parameter 2 of RB1-pp dephosphorylation	2.11	Calibrated
(53) k_{pro}	Basal proliferation rate	1.2×10^{-4}/hour	Calibrated
(54) $k_{proRB1pp}$	Proliferation rate increased by RB1-pp	266.30	Calibrated
(55) $p_{proRB1pp_1}$	Parameter 1 of proliferation rate increased by RB1-pp	6.01μM	Calibrated
(56) $p_{proRB1pp_2}$	Parameter 2 of proliferation rate increased by RB1-pp	6	Fixed
(57) $k_{carrying}$	Carrying capacity	37.39a.u.	Calibrated
(58) $E2_{dep1}$	Estrogen concentration in E2 deprivation media 0 to 3days	9.67×10^{-5}μM (2)	Calibrated
(59) $E2_{dep2}$	Estrogen concentration in E2 deprivation media 3 to 7days	2.30×10^{-5}μM	Calibrated
(60) $E2$	Estrogen concentration	0.01μM	Fixed
(61) ICI	ICI 182,780 concentration	0.5μM	Fixed
(62) palbo	Palbociclib concentration	1μM	Fixed

The culture media, including any drugs, is changed at T = 0 and T = 3d for the experiments (E2control, E2 deprivation, +E2+ICI and E2 deprivation+ICI, +E2+palbo and E2 deprivation+palbo), so the longest period without resupplying the drugs is 4 days. In the model, the drug concentration is assumed to be constant throughout the experiment. There is no data we are aware of for the half-life of either palbociclib or fulvestrant in our in-vitro culture conditions. Most data is for the plasma half-life or terminal half-life in-vivo, which are not applicable to our case as they are determined primarily by processing in the liver and excretion through the kidneys. There is some data for the in-vitro stability of these drugs in human plasma, which is somewhat akin to our conditions. Palbociclib

shows less than 5% degradation in human plasma at room temperature over 3 days [72]. Fulvestrant shows no degradation in human plasma at room temperature over 7 hours [73]. Based on this data, we believe the half-life in our system is sufficiently long that assuming a constant level of drug is a reasonable approximation.

Model Equations

$$\frac{dER}{dt} = k_{ER} - kd_{ER} \times ER \ (1)$$
$$-kb_{E2ER} \times E2 \times ER + kub_{E2ER} \times E2ER \ (2)$$
$$-kb_{ICIER} \times ICI \times ER + kub_{ICIER} \times ICIER \ (3)$$

(1) Translation and degradation of ERα

(2) Binding and unbinding between ERα and E2

(3) Binding and unbinding between ERα and ICI 182,780

$$\frac{dE2ER}{dt} = -kd_{E2ER} \times E2ER \ (4)$$
$$+kb_{E2ER} \times E2 \times ER - kub_{E2ER} \times E2ER \ (5)$$

(4) Degradation of E2:ER

(5) Binding and unbinding between ERα and E2

$$\frac{dICIER}{dt} = kb_{ICIER} \times ICI \times ER - kub_{ICIER} \times ICIER \ (6)$$
$$-kd_{ICIER} \times ICIER \ (7)$$

(6) Binding and unbinding between ICI 182,780 and ERα

(7) Degradation of ICIER

$$\frac{dcyclinD1}{dt} = -kd_{cyclinD1} \times cyclinD1 \ (8)$$
$$+k_{cyclinD1} \times \left(1 + k_{cyclinD1E2ER} \times \frac{E2ER^{p_{cyclinD1E2ER_2}}}{{p_{cyclinD1E2ER_1}}^{p_{cyclinD1E2ER_2}} + E2ER^{p_{cyclinD1E2ER_2}}}\right) \ (9)$$
$$-kb_{cyclinD1p21} \times cyclinD1 \times p21 + kub_{cyclinD1p21} \times cyclinD1p21 \ (10)$$
$$-kb_{cylinD1palbo} \times cyclinD1 \times palbo + kub_{cyclinD1palbo} \times cyclinD1palbo \ (11)$$

(8) Degradation of cyclinD1

(9) Basal translation of cyclinD1 and the increased translation by E2:ER

(10) Binding and unbinding between cyclinD1 and p21

(11) Binding and unbinding between cyclinD1 and palbociclib

$$\frac{dcyclinD1p21}{dt} = -kd_{cyclinD1} \times cyclinD1p21 \ (12)$$
$$+kb_{cyclinD1p21} \times cyclinD1 \times p21 - kub_{cyclinD1p21} \times cyclinD1p21 \ (13)$$

(12) Degradation of p21 bound cyclinD1

(13) Binding and unbinding between cyclinD1 and p21

$$\frac{dcyclinD1palbo}{dt} = -kd_{cyclinD1} \times cyclinD1palbo \ (14)$$
$$+kb_{cyclinD1palbo} \times cyclinD1 \times palbo - kub_{cyclinD1palbo} \times cyclinD1palbo \ (15)$$

(14) Degradation of palbociclib bound cyclinD1

(15) Binding and unbinding between cyclinD1 and palbociclib

$$\frac{dcMyc}{dt} = -kd_{cMyc} \times cMyc \text{ (16)}$$
$$+k_{cMyc} \times (1 + k_{cMycE2ER} \times \frac{E2ER^{p_{cMycE2ER_2}}}{{p_{cMycE2ER_1}}^{p_{cMycE2ER_2}} + E2ER^{p_{cMycE2ER_2}}} \text{ (17)}$$
$$+k_{cMycRB1pp} \times \frac{RB1pp^{p_{cMycRB1pp_2}}}{{p_{cMycRB1pp_1}}^{p_{cMycRB1pp_2}} + RB1pp^{p_{cMycRB1pp_2}}}) \text{ (18)}$$

(16) Degradation of c-Myc

(17) Basal translation of c-Myc and the increased translation by E2:ER

(18) Increased translation of c-Myc by RB1-pp

$$\frac{dp21}{dt} = -kd_{p21} \times p21 \text{ (19)}$$
$$+k_{p21} \times \frac{{p_{p21cMyc_1}}^{p_{p21cMyc_2}}}{{p_{p21cMyc_1}}^{p_{p21cMyc_2}} + cMyc^{p_{p21cMyc_2}}} \text{ (20)}$$
$$-kb_{cyclinD1p21} \times cyclinD1 \times p21 + kub_{cyclinD1p21} \times cyclinD1p21 \text{ (21)}$$
$$-kb_{cyclinEp21} \times cyclinE \times p21 + kub_{cyclinEp21} \times cyclinEp21 \text{ (22)}$$

(19) Degradation of p21

(20) Basal translation and the inhibition of translation by c-Myc

(21) Binding and unbinding between cyclinD1 and p21

(22) Binding and unbinding between cyclinE and p21

$$\frac{dcyclinE}{dt} = k_{cyclinE} - kd_{cyclinE} \times cyclinE \text{ (23)}$$
$$-kb_{cyclinEp21} \times cyclinE \times p21 + kub_{cyclinEp21} \times cyclinEp21 \text{ (24)}$$

(23) Translation and degradation of cyclinE

(24) Binding and unbinding between cyclinE and p21

$$\frac{dcyclinEp21}{dt} = -kd_{cyclinE} \times cyclinEp21 \ (25)$$
$$+kb_{cyclinEp21} \times cyclinE \times p21 - kub_{cyclinEp21} \times \ cyclinEp21 \ (26)$$

(25) Degradation of p21 bound cyclinE

(26) Binding and unbinding between cyclinE and p21

$$\frac{dRB1}{dt} = k_{RB1} - kd_{RB1} \times RB1 \ (27)$$
$$+k_{RB1RB1pp} \times \frac{RB1pp^{p_{RB1RB1pp_2}}}{p_{RB1RB1pp_1}{}^{p_{RB1RB1pp_2}} + RB1pp^{p_{RB1RB1pp_2}}} \ (28)$$
$$-k_{RB1cyclinD1} \times cyclinD1 \times \frac{RB1^{p_{cyclinD1RB1_2}}}{p_{cyclinD1RB1_1}{}^{p_{cyclinD1RB1_2}} + RB1^{p_{cyclinD1RB1_2}}} \ (29)$$
$$+k_{RB1pdepho} \times \frac{RB1p^{p_{RB1p_2}}}{p_{pRB1p_1}{}^{p_{RB1p_2}} + RB1p^{p_{RB1p_2}}} \ (30)$$

(27) Degradation of RB1 and basal translation

(28) Increased translation by E2F, modeled as proportional to RB1-pp

(29) Phosphorylation of RB1 by cyclinD1

(30) Dephosphorylation of RB1-p

$$\frac{dRB1p}{dt} = -kd_{RB1p} \times RB1p \ (31)$$
$$+k_{RB1cyclinD1} \times cyclinD1 \times \frac{RB1^{p_{cyclinD1RB1_2}}}{p_{cyclinD1RB1_1}{}^{p_{cyclinD1RB1_2}} + RB1^{p_{cyclinD1RB1_2}}} \ (32)$$
$$-k_{RB1pdepho} \times \frac{RB1p^{p_{RB1p_2}}}{p_{RB1p_1}{}^{p_{RB1p_2}} + RB1p^{p_{RB1p_2}}} \ (33)$$
$$-k_{RB1pcyclinE} \times cyclinE \times \frac{RB1p^{p_{cyclinERB1p_2}}}{p_{cyclinERB1p_1}{}^{p_{cyclinERB1p_2}} + RB1p^{p_{cyclinERB1p_2}}} \ (34)$$
$$+k_{RB1ppdepho} \times \frac{RB1pp^{p_{RB1pp_2}}}{p_{RB1pp_1}{}^{p_{RB1pp_2}} + RB1pp^{p_{RB1pp_2}}} \ (35)$$

(31) Degradation of RB1-p

(32) Phosphorylation of RB1 by cyclinD1

(33) Dephosphorylation of RB1-p

(34) Phosphorylation of RB1-p by cyclinE

(35) Dephosphorylation of RB1-pp

$$\frac{dRB1pp}{dt} = -kd_{RB1pp} \times RB1pp \ (36)$$
$$+k_{RB1pcyclinE} \times cyclinE \times \frac{RB1p^{p_{cyclinERB1p_2}}}{{p_{cyclinERB1p_1}}^{p_{cyclinERB1p_2}} + RB1p^{p_{cyclinERB1p_2}}} \ (37)$$
$$-k_{RB1ppdepho} \times \frac{RB1pp^{p_{RB1pp_2}}}{{p_{RB1pp_1}}^{p_{RB1pp_2}} + RB1pp^{p_{RB1pp_2}}} \ (38)$$

(36) Degradation of RB1-pp

(37) Phosphorylation of RB1-p by cyclinE

(38) Dephosphorylation of RB1-pp

$$\frac{dcell}{dt} = k_{pro} \times (1 + k_{proRB1pp} \times \frac{RB1pp^{p_{proRB1pp_2}}}{{p_{proRB1pp_1}}^{p_{proRB1pp_2}} + RB1pp^{p_{proRB1pp_2}}}) \times cell \times (1 - \frac{cell}{k_{carrying}}) \ (39)$$

(39) Basal proliferation and the increased proliferation by RB1-pp

To model resistance, we added the following equation:

$$\frac{dres}{dt} = par1_{res} \times palbo - par2_{res} \times res \ \ (40)$$

and added one term to the cyclinD1 equation:

$$\frac{dcyclinD1}{dt} = -kd_{cyclinD1} \times cyclinD1 \ (8)$$
$$+k_{cyclinD1} \times \left(1 + k_{cyclinD1E2ER} \times \frac{E2ER^{p_{cyclinD1E2ER_2}}}{{p_{cyclinD1E2ER_1}}^{p_{cyclinD1E2ER_2}} + E2ER^{p_{cyclinD1E2ER_2}}}\right) \ (9)$$
$$-kb_{cyclinD1p21} \times cyclinD1 \times p21 + kub_{cyclinD1p21} \times cyclinD1p21 \ (10)$$
$$-kb_{cylinD1palbo} \times cyclinD1 \times palbo + kub_{cyclinD1palbo} \times cyclinD1palbo \ (11)$$
$$+par3_{res} \times \frac{res^{par5_{res}}}{{par4_{res}}^{par5_{res}} + res^{par5_{res}}} \ (41)$$

$par1_{res} = 1e^{-4},\ par2_{res} = 1e^{-3}, par3_{res} = 0.819, par4_{res} = 0.06, par5_{res} = 4.87$

Model Summary

The mathematical model contains 14 ordinary differential equations (ODEs) and has 62 parameters. It is implemented in MATLAB (MathWorks, Inc., Massachusetts, United States). The synthesis, degradation, phosphorylation, dephosphorylation, association and dissociation reactions are modeled by mass action laws and Hill functions. ICI 182,780 and palbociclib effects are modeled by competitive binding to ERα [50, 60] or cyclinD1, representing cyclinD1:Cdk4/6 [74]. The ODEs are solved numerically by the *ode23tb* function. RB1 has an important role in the G1-S transition and it is inactivated by phosphorylation [75]. A total of over 15 phosphorylation sites are found on *RB1* (i.e. T5, S249, S252, T356, T373, S567, S608, S612, S780, S788, S795, S807, S811, T821, T826) [76]. In late G1, cyclinE:Cdk2 kinase mediates the phosphorylation of RB1 on S612 and T373 relieving RB1's repression of E2F activity [76, 77]. As cyclinD1:Cdk4/6 kinases hypophosphorylate RB1 in G1 and cyclinE:Cdk2 kinase hyperphosphorylates RB1-p in late G1 [78-80], we chose one form of phosphorylated RB1 (S612) to represent the phosphorylation status of RB1 by cyclinE:Cdk2 kinase.

Data Normalization

Protein levels were measured by Western blotting at time points 0h, 4h, 1d, 3d, 6d and 7d for the E2 deprivation, +E2+ICI and –E2+ICI conditions, and at 0h, 1d, 3d, 6d and 7d for the +E2+palbo and –E2+palbo conditions. The blots for the treatment conditions are normalized to actin and β-tubulin, to account for loading variation, normalized to time 0h, to account for antibody affinity variations, and normalized to the (normalized) E2 control values for each species at each time point, to account for variations in the expression levels of species in the cells used for a given experiment. Since the control cells are not subject to treatment perturbations (they continue to be grown in E2), the asynchronous population would ideally have constant expression levels of the various molecules. This is not the case

in our experiments as there are frequently transients during the first few time points, perhaps due to the shock of reattachment upon plating before time 0. Furthermore, after 3 days the control cells begin to approach confluence, while the cells under treatment conditions do not begin to approach confluence until 7 days. At confluence, paracrine signaling and contact inhibition suppress proliferation via cell cycle arrest [81-84]. To avoid the confounding effect of confluence on the control cells, we assume the expression levels of the species in the control cells after 3 days are the same as their levels at the 3d timepoint and use the 3d levels for normalization of the future timepoints of the treated cells. This approach allows our normalization to deal with the initial transients in expression levels while not confounding the normalization of timepoints after 3 days with the effects of confluence on the control cells.

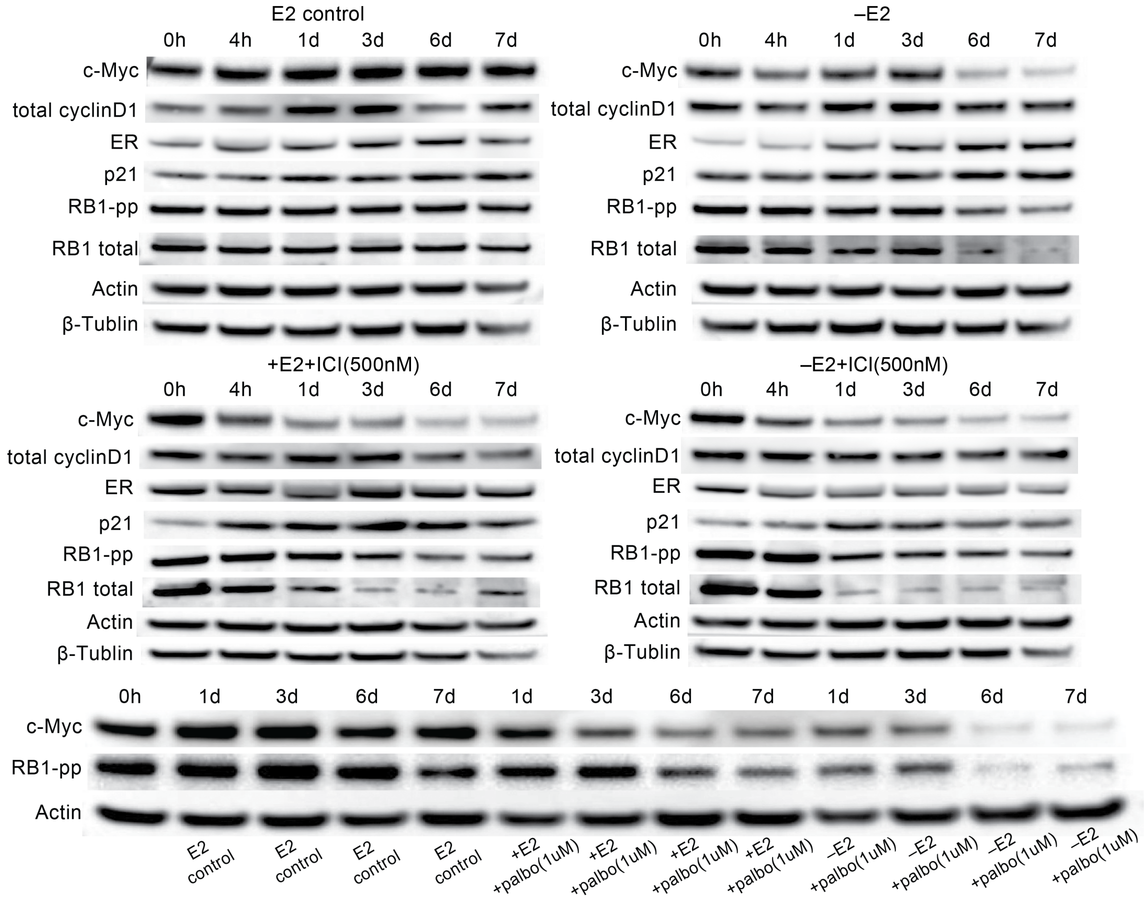

Fig. S1: Representative western blot data shown here for different treatment conditions. All experiments were done in triplicate.

Local Sensitivity Analysis

Local sensitivity analysis concerns the sensitivity of a model output to changes in the input parameters [85]. The local sensitivities of the seven measured output variables are calculated with respect to the parameters at day 7 timepoint [86].

$$s_{ijk} = \frac{\partial \log(X_{ij})}{\partial \log(P_k)} = \frac{\partial X_{ij}}{\partial P_k} \frac{P_K}{X_{ij}} \quad (42)$$

where s_{ijk} is the local sensitivity value, which is the derivative of output X_{ij} with respect to parameter P_k multiplied by the ratio P_k/X_{ij}. It represents the relative change in the model output induced by a small relative change in a parameter. In the equation, i indexes the outputs (protein level or cell number), j indexes the timepoints, and k indexes the parameters.

s_{ijk} is approximated by the second order central finite difference. Each parameter is individually varied by ±5% of its value. Therefore,

$$s_{ijk} \approx \frac{X_{ij}(P_k + 5\% \times P_k) - X_{ij}(P_k - 5\% \times P_k)}{10\% \times P_k} \frac{P_k}{X_{ij}(P_k)}$$

$$= \frac{X_{ij}(P_k + 5\% \times P_k) - X_{ij}(P_k - 5\% \times P_k)}{10\% \times X_{ij}(P_k)} \quad (43)$$

All parameters that were varied to fit the data were selected for local sensitivity analysis, while the fixed parameters were excluded. The 44 parameters, from top to bottom in Fig 6 and Fig. S2, are: k_{ER}, kb_{E2ER}, kb_{ICIER}, kd_{ICIER}, $k_{cyclinD1}$, $k_{cyclinD1E2ER}$, $p_{cyclinD1E2ER_1}$, $p_{cyclinD1E2ER_2}$, $kb_{cyclinD1p21}$, $kb_{cyclinD1palbo}$, k_{cMyc}, $k_{cMycE2ER}$, $p_{cMycE2ER_1}$, $p_{cMycE2ER_2}$, $k_{cMycRB1pp}$, $p_{cMycRB1pp_1}$, $p_{cMycRB1pp_2}$, k_{p21}, $p_{p21cMyc_1}$, $p_{p21cMyc_2}$, $k_{cyclinE}$, $kb_{cyclinEp21}$, k_{RB1}, $k_{RB1RB1pp}$, $p_{RB1RB1pp_1}$, $p_{RB1RB1pp_2}$, $k_{RB1cyclinD1}$, $k_{RB1pdepho}$, $k_{RB1pcyclinE}$, $k_{RB1ppdepho}$, $p_{cyclinD1RB1_1}$, $p_{cyclinD1RB1_2}$, $p_{RB1pdepho_1}$, $p_{RB1pdepho_2}$ $p_{cyclinERB1p_1}$, $p_{cyclinERB1p_2}$, $p_{RB1ppdepho_1}$, $p_{RB1ppdepho_2}$, k_{pro}, $k_{proRB1pp}$, $k_{proRB1pp_1}$, $k_{carrying}$, $E2_{dep1}$, $E2_{dep2}$.

Local sensitivity of parameters

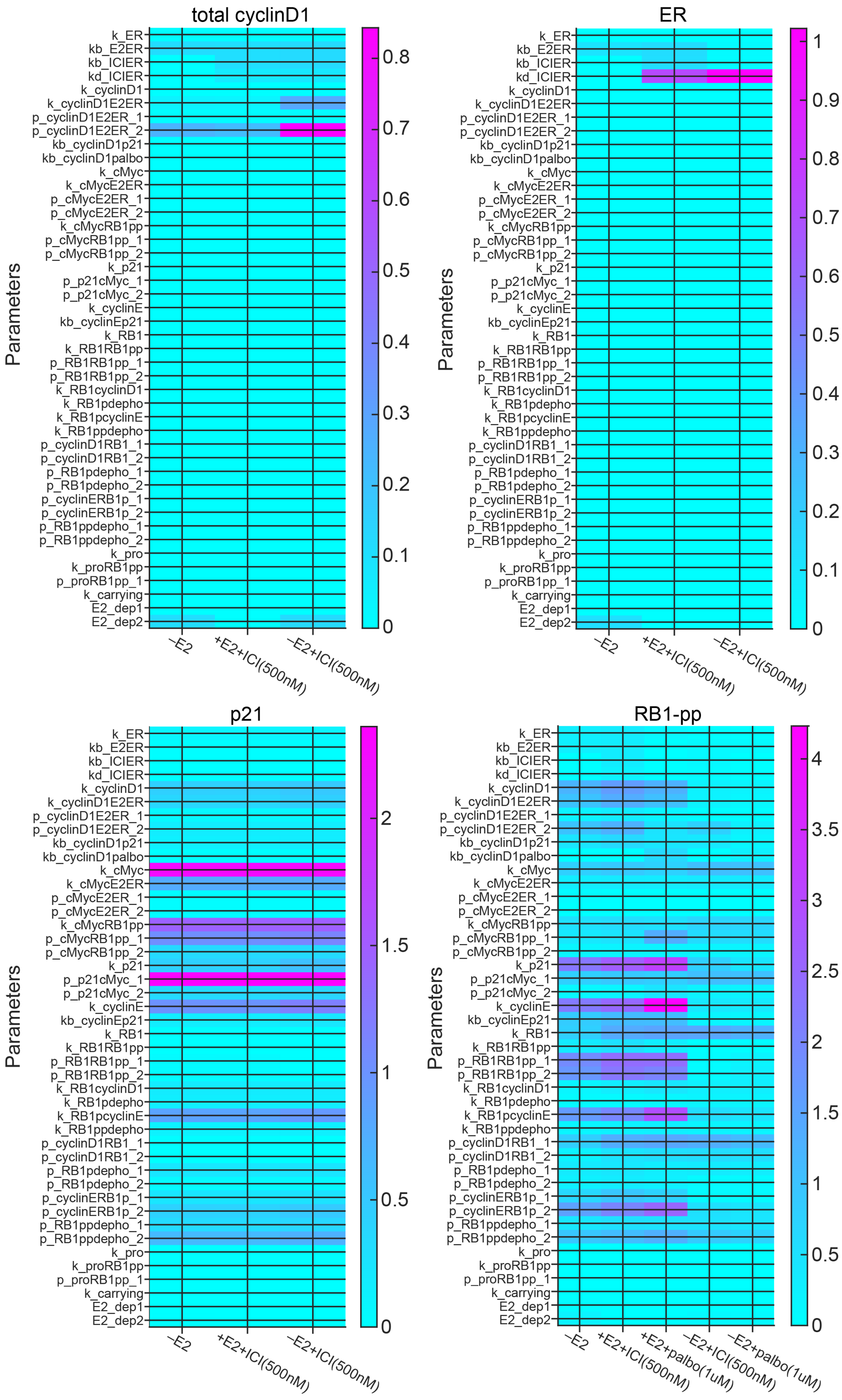

Fig. S2: Local Sensitivity of total cyclinD1, total ER, total p21 and RB1-pp. Sensitivity of each protein at the 7d timepoint with respect to all variable parameters.

Variation of the Cohort Parameters and Plots of the Individual Trajectories

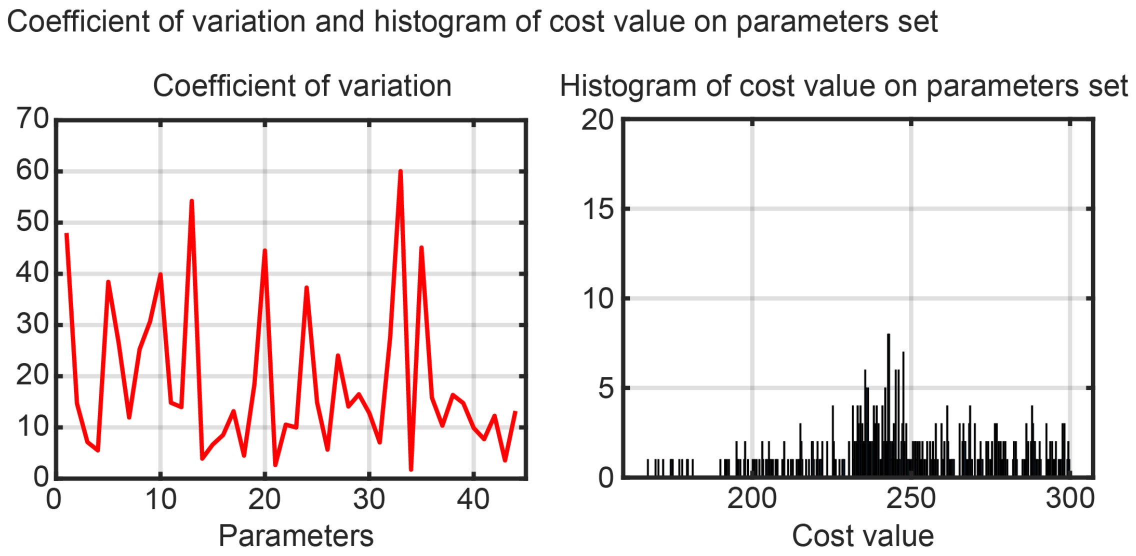

Fig. S3: Coefficients of variation and histogram of cost values for parameter sets in the simulation cohort. Ordering of parameters corresponds to that in the sensitivity plots.

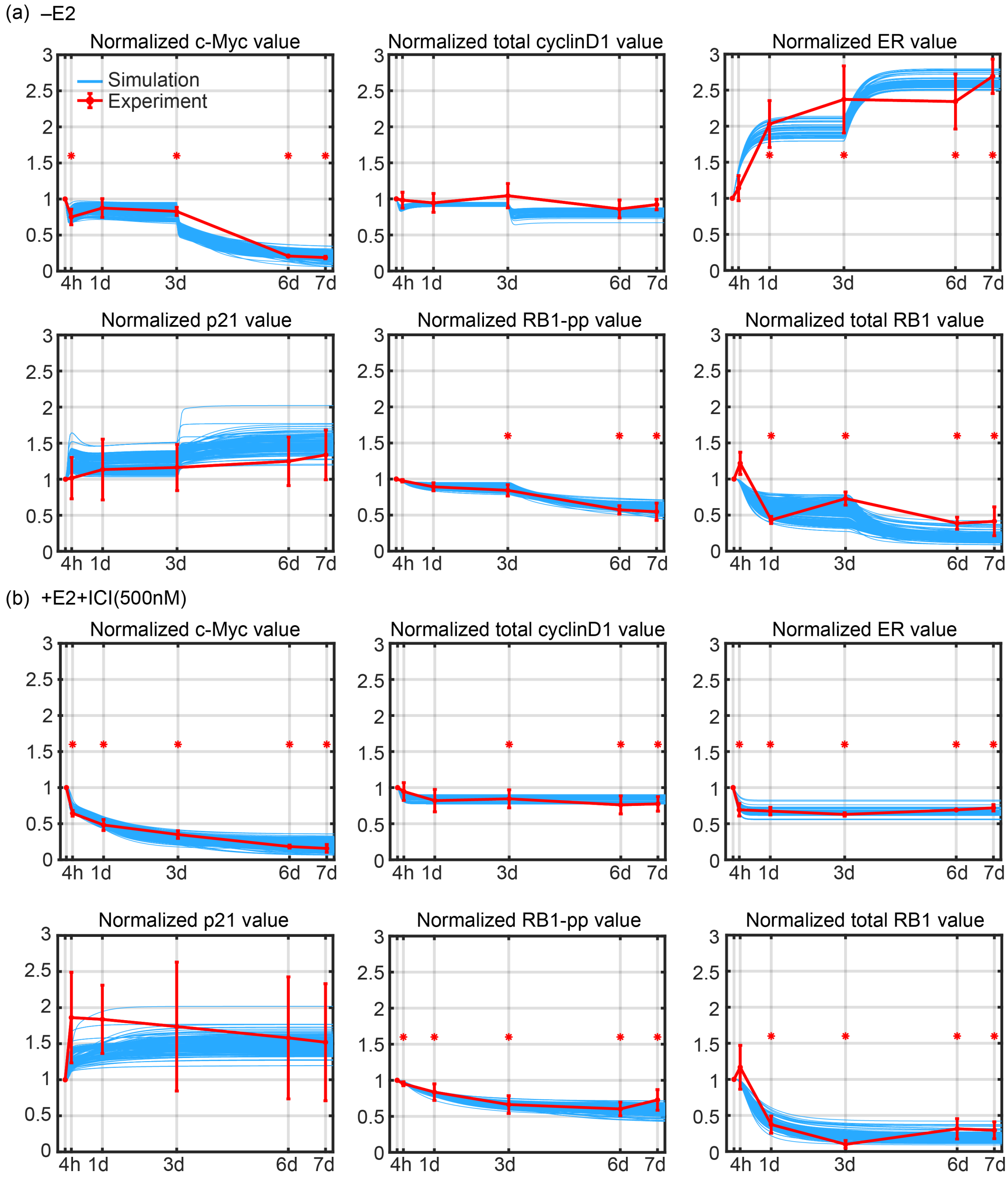

Fig. S4: All cohort trajectories plotted with experimental protein data that was used to fit the model. (a) –E2 condition. (b) +E2+ICI(500nM) condition.

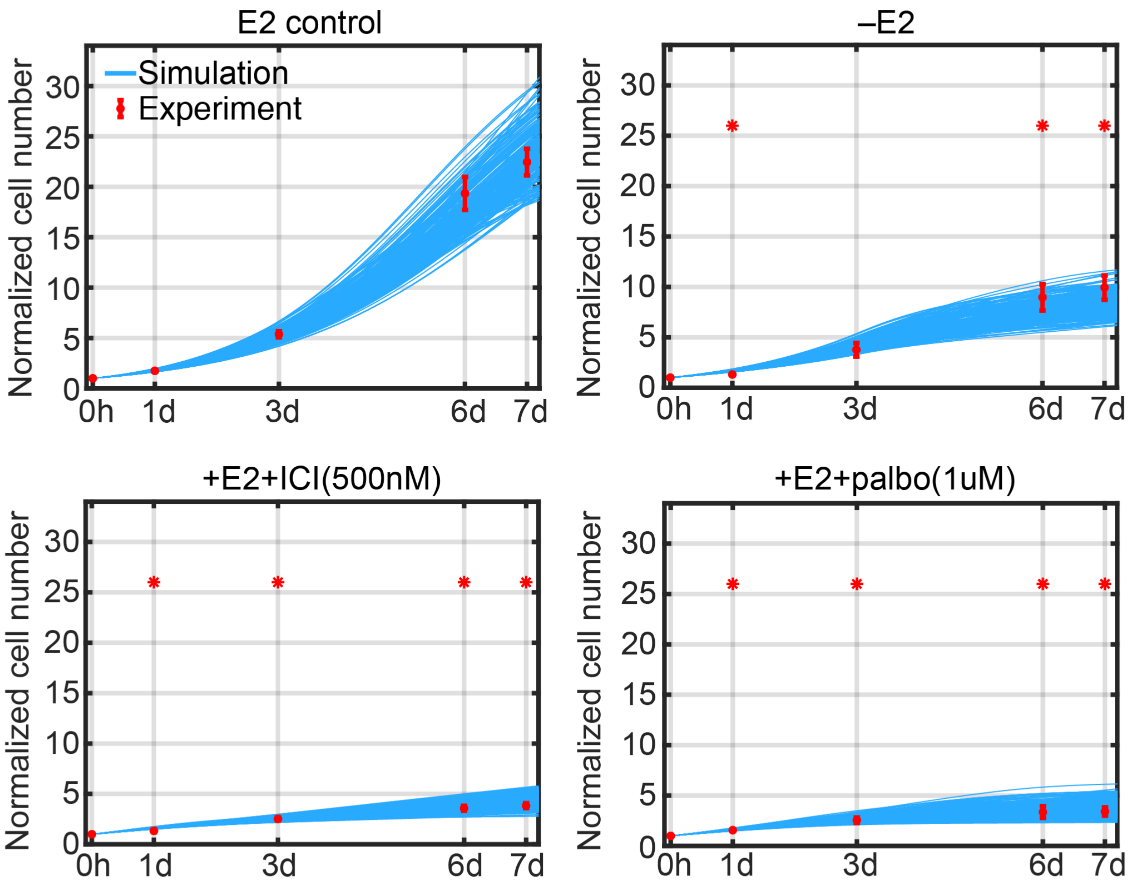

Fig. S5: All cohort trajectories plotted with experimental proliferation data that was used to fit the model.

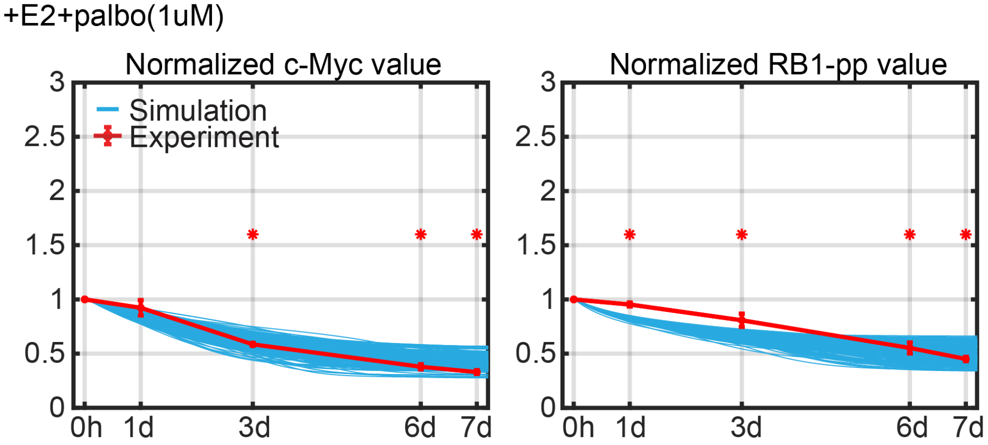

Fig. S6: All cohort trajectories plotted with experimental protein data used to fit the parameters involving palbociclib in the model.

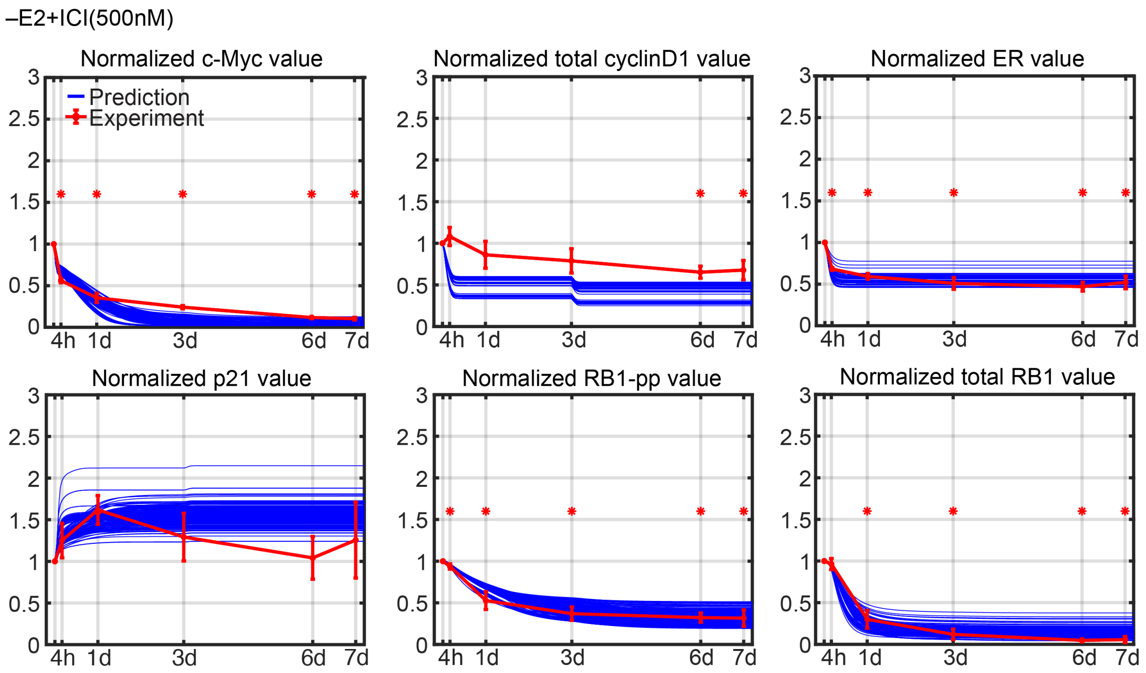

Fig. S7: All cohort trajectory predictions for the –E2+ICI(500nM) case plotted with experimental protein validation data.

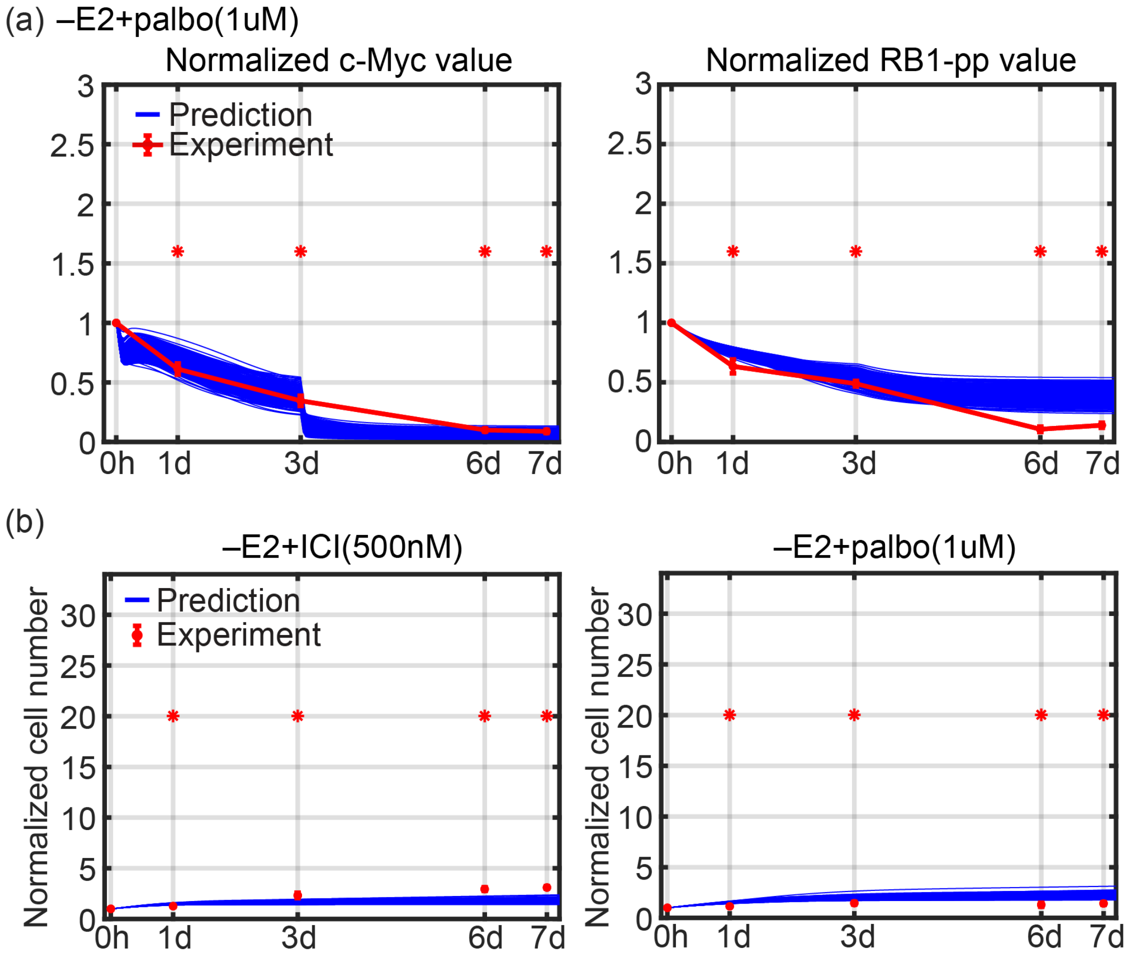

Fig. S8: All cohort trajectory predictions plotted with the experimental validation data. (a) Protein predictions for the –E2+palbociclib(1uM) case. (b) Proliferation predictions for the –E2+ICI(500nM) and –E2+palbociclib(1uM) cases.

Synergy Analysis of the Combination Treatments

Fig. S9 shows the synergy scores of the combination treatments –E2+ICI(500nM) and –E2+palbociclib(1uM) for proliferation at different timepoints. The HSA and Bliss synergy scores are calculated using the R package SynergyFinder [87]. The score, in percent, is equal to the percent

inhibition of the combination therapy minus the expected inhibition. For HSA the expected inhibition is the highest percent inhibition of the two mono therapies, and for Bliss the expected inhibition is the sum of the percent inhibition of each mono therapy minus the product of the percent inhibitions of the two therapies. Thus, a positive score represents a synergistic effect. There is no significant synergy, and some antagonism indicated in the experimental results for the –E2+ICI(500nM) combination therapy. The simulations show some synergy for this case because they slightly under-predict the true proliferation of the combination. For the case of the –E2+palbociclib(1uM) combination, there is some indication of synergy in the experimental results and slightly less in the simulation results due to the slight overprediction of proliferation for the combination in the simulations. A synergistic effect between –E2 and palbociclib is consistent with the use of palbociclib in conjunction with endocrine therapies in the clinic [88-90].

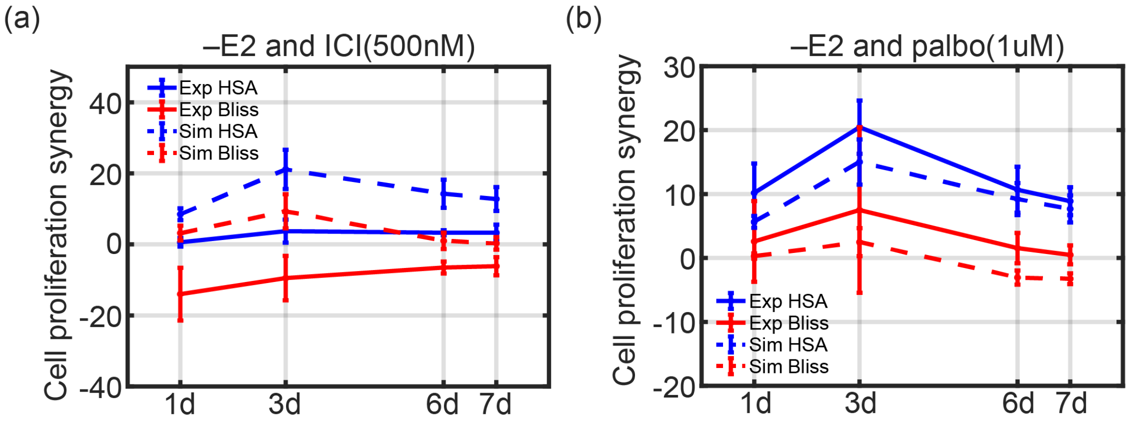

Fig. S9: HSA and Bliss cell proliferation synergy scores of –E2+ICI(500nM) and –E2+palbociclib(1uM) treatments. The scores of the experimental results are shown with solid lines (mean value ± s.d., n = 3). The scores of the simulation results are shown with dashed lines (mean value ± s.d., n = 400).

References

1. Chia YH, Ellis MJ, Ma CX. Neoadjuvant endocrine therapy in primary breast cancer: Indications and use as a research tool. Br J Cancer. 2010;103(6):759–764. (doi:10.1038/sj.bjc.6605845)

2. Tremont A, Lu J, Cole JT. Endocrine Therapy for Early Breast Cancer: Updated Review. Ochsner J. 2017; 17:405–411.

3. Levine MN, Whelan T. Adjuvant Chemotherapy for Breast Cancer — 30 Years Later. N Engl J Med. 2006;355(18):1920–1922. (doi:10.1056/NEJMe068204)

4. Smith I, Yardley D, Burris H, De Boer R, Amadori D, McIntyre K, et al. Comparative efficacy and safety of adjuvant letrozole versus anastrozole in postmenopausal patients with hormone receptor-positive, node-positive early breast cancer: Final results of the randomized phase III femara versus anastrozole clinical evaluation. J Clin Oncol. 2017;35(10):1041–1048. (doi:10.1200/JCO.2016.69.2871)

5. Ma CX, Reinert T, Chmielewska I, Ellis MJ. Mechanisms of aromatase inhibitor resistance. Nat Rev Cancer. 2015;15(5):261–275. (doi:10.1038/nrc3920)

6. Lin NU, Winer EP. Advances in adjuvant endocrine therapy for postmenopausal women. J Clin Oncol. 2008;26(5):798–805. (doi:10.1200/JCO.2007.15.0946)

7. Leo A Di, Jerusalem G, Petruzelka L, Torres R, Bondarenko IN, Khasanov R, et al. Final overall survival: Fulvestrant 500 mg vs 250 mg in the randomized confirm trial. J Natl Cancer Inst. 2014;106(1):1–7. (doi:10.1093/jnci/djt337)

8. Seruga B, Tannock IF. Up-front use of aromatase inhibitors as adjuvant therapy for breast cancer: The emperor has no clothes. J Clin Oncol. 2009;27(6):840–842. (doi:10.1200/JCO.2008.19.5594)

9. Wakeling AE, Dukes M, Bowler J. A potent specific pure antiestrogen with clinical potential. Cancer Res. 1991 Aug; 51(15): 3867–3873.

10. Wakeling AE. The future of new pure antiestrogens in clinical breast cancer. Breast Cancer Res Treat. 1993 Jan; 25(1): 1–9. (doi:10.1007/BF00662395)

11. Wittmann BM, Sherk A, McDonnell DP. Definition of functionally important mechanistic differences among selective estrogen receptor down-regulators. Cancer Res. 2007; 67(19): 9549–9560. (doi:10.1158/0008-5472.CAN-07-1590)

12. Farzaneh S, Zarghi A. Estrogen receptor ligands: A review (2013–2015). Sci Pharm. 2016; 84(3): 409–427. (doi:10.3390/scipharm84030409)

13. McDonnell DP, Norris JD. Connection and regulation of the human estrogen receptor. Science. 2002; 296(5573):1642–1644. (doi:10.1126/science.1071884)

14. Musgrove EA, Sutherland RL. Biological determinants of endocrine resistance in breast cancer. Nat Rev Cancer. 2009; 9(9): 631–643. (doi:10.1038/nrc2713)

15. Vrtačnik P, Ostanek B, Mencej-Bedrač S, Marc J. The many faces of estrogen signaling. Biochem Medica. 2014; 24(3): 329–242. (doi:10.11613/BM.2014.035)

16. Prall OW, Rogan EM, Musgrove E a, Watts CK, Sutherland RL. c-Myc or cyclin D1 mimics estrogen effects on cyclin E-Cdk2 activation and cell cycle reentry. Mol Cell Biol. 1998; 18(8):4499–4508. (doi:10.1128/MCB.18.8.4499)

17. Butt AJ, McNeil CM, Musgrove EA, Sutherland RL. Downstream targets of growth factor and oestrogen signalling and endocrine resistance: The potential roles of c-Myc, cyclin D1 and cyclin E. Endocr Relat Cancer. 2005; 12(SUPPL. 1): 47–60. (doi:10.1677/erc.1.00993)

18. Dubik D, Dembinski TC, Shiu RPC. Stimulation of c-myc oncogene expression associated with estrogen-induced proliferation of human breast cancer cells. Cancer Res. 1987; 47:6517–6521.

19. Dubik D, Shiu RPC. Transcriptional regulation of c-myc oncogene expression by estrogen in hormone-responsive human breast cancer cells. J Biol Chem. 1988; 263(25): 12705–12708.

20. Dubik D, Shiu RP. Mechanism of estrogen activation of c-myc oncogene expression. Oncogene. 1992 Aug; 7(8): 1587–1594.

21. Hanson KD, Shichiri M, Follansbee MR, Sedivy JM. Effects of c-myc expression on cell cycle progression. Mol Cell Biol. 1994; 14(9): 5748–5755. (doi:10.1128/MCB.14.9.5748)

22. Nass SJ, Dickson RB. Defining a role for c-Myc in breast tumorigenesis. Breast Cancer Research and Treatment. 1997. p. 1–22. (doi:10.1023/A:1005858611585)

23. Bretones G, Delgado MD, León J. Myc and cell cycle control. BBA - Gene Regul Mech. Elsevier B.V.; 2015; 1849(5): 506–516. (doi:10.1016/j.bbagrm.2014.03.013)

24. Peukert K, Staller P, Schneider A, Carmichael G, Hänel F, Eilers M. An alternative pathway for gene regulation by Myc. EMBO J. 1997; 16(18): 5672–5686. (doi:10.1093/emboj/16.18.5672)

25. Möröy T, Saba I, Kosan C. The role of the transcription factor Miz-1 in lymphocyte development and lymphomagenesis-Binding Myc makes the difference. Semin Immunol. 2011; 23(5): 379–387. (doi:10.1016/j.smim.2011.09.001)

26. Yang W, Shen J, Wu M, Arsura M, Fitzgerald M, Suldan Z, et al. Repression of transcription of the p27Kip1 cyclin-dependent kinase inhibitor gene by c-Myc. Oncogene. 2001;(January): 1688–1702. (doi:10.1038/sj.onc.1204245)

27. Chandramohan V, Mineva ND, Burke B, Jeay S, Wu M, Shen J, et al. c-Myc represses FOXO3a-mediated transcription of the gene encoding the p27Kip1cyclin dependent kinase inhibitor. J Cell Biochem. 2008; 104(6): 2091–2106. (doi:10.1002/jcb.21765)

28. Sabbah M, Courilleau D, Mester J, Redeuilh G. Estrogen induction of the cyclin D1 promoter: Involvement of a cAMP response-like element. Proc Natl Acad Sci U S A. 1999; 96(20):11217–11222. (doi:10.1073/pnas.96.20.11217)

29. Eeckhoute J, Carroll JS, Geistlinger TR, Torres-arzayus MI, Brown M. A cell-type-specific transcriptional network required for estrogen regulation of cyclin D1 and cell cycle progression in breast cancer. Genes Dev. 2006; 1:2513–2526. (doi:10.1101/gad.1446006)

30. Castro-Rivera E, Samudio I, Safe S. Estrogen Regulation of Cyclin D1 Gene Expression in ZR-75 Breast Cancer Cells Involves Multiple Enhancer Elements. J Biol Chem. 2001; 276(33): 30853–30861. (doi:10.1074/jbc.M103339200)

31. Daksis JI, Lu RY, Facchini LM, Marhin WW, Penn LJ. Myc induces cyclin D1 expression in the absence of de novo protein synthesis and links mitogen-stimulated signal transduction to the cell cycle. Oncogene. 1994 Dec; 9(12): 3635–3645.

32. Jansen-durr P, Meichlet A, Steinert P, Paganot M, Finket K, Botz J, et al. Differential modulation of cyclin gene expression by MYC. Proc Natl Acad Sci U S A. 1993; 90(April): 3685–3689. (doi:10.1073/pnas.90.8.3685)

33. Philipp A, Schneider A, Vasrik I, Finke K, Xiong YUE, Beach D, et al. Repression of Cyclin D1: a Novel Function of MYC. Mol Cell Biol. 1994; 14(6): 4032–4043. (doi:10.1128/MCB.14.6.4032)

34. Huerta M, Munoz R, Tapia R, Soto-Reyes E, Ramirez L, Recillas-Targa F, Gonzalez-Mariscal L, Lopez-Bayghen E. Cyclin D1 Is Transcriptionally Down-Regulated by ZO-2 via an E Box and the Transcription Factor c-Myc. Mol Biol Cell. 2007; 18(December): 4826–4836. (doi:10.1091/mbc.e07-02-0109)

35. Hermeking H, Funk JO, Reichert M, Ellwart JW, Eick D. Abrogation of p53-induced cell cycle arrest by c-Myc: evidence for an inhibitor of p21WAF1/CIP1/SDI1. Oncogene. 1995 Oct; 11(7): 1409–1415.

36. Huang TS, Duyster J, Wang JY. Biological response to phorbol ester determined by alternative G1 pathways. Proc Natl Acad Sci U S A; 1995 May; 92(11): 4793–4797. (doi:10.1073/pnas.92.11.4793)

37. Solomon DL, Philipp A, Land H, Eilers M. Expression of cyclin D1 mRNA is not upregulated by Myc in rat fibroblasts. Oncogene; 1995 Nov; 11(9): 1893–1897.

38. Watts CKW, Prall OWJ, Carroll JS, Wilcken NRC, Rogan EM, Musgrove EA, et al. Antiestrogens and the Cell Cycle. In: Jordan VC, Furr BJA, editors. Hormone Therapy in Breast and Prostate Cancer. Totowa, NJ: Humana Press; 2009. p. 17–45. (doi:10.1007/978-1-59259-152-7_2)

39. Sweeney KJ, Swarbrick A, Sutherland RL, Musgrove EA. Lack of relationship between CDK activity and G1 cyclin expression in breast cancer cells. Oncogene. 1998 Jun 3;16:2865. (doi:10.1038/sj.onc.1201814)

40. Sherr CJ. D-type cyclins. Trends Biochem Sci. 1995; 20(5): 187–190. (doi:10.1016/S0968-0004(00)89005-2)

41. Sherr CJ, Roberts JM. Inhibitors of mammalian cyclin-dependent kinases. Genes Dev. 1995; 1149–1163. (doi:10.1101/gad.9.10.1149)

42. Pei X-H, Xiong Y. Biochemical and cellular mechanisms of mammalian CDK inhibitors: a few unresolved issues. Oncogene. 2005; 24(17): 2787–2795. (doi:10.1038/sj.onc.1208611)

43. Musgrove EA, Caldon CE, Barraclough J, Stone A, Sutherland RL. Cyclin D as a therapeutic target in cancer. Nat Publ Gr. 2011; 11(8): 558–572. (doi:10.1038/nrc3090)

44. Skildum AJ, Mukherjee S, Conrad SE. The cyclin-dependent kinase inhibitor p21WAF1/Cip1 is an antiestrogen-regulated inhibitor of Cdk4 in human breast cancer cells. J Biol Chem. 2002; 277(7): 5145–5152. (doi:10.1074/jbc.M109179200)

45. Foster JS, Wimalasena J. Estrogen regulates activity of cyclin-dependent kinases and retinoblastoma protein phosphorylation in breast cancer cells. Mol Endocrinol. 1996; 10(5): 488–498. (doi:10.1210/mend.10.5.8732680)

46. Planas-Silva MD, Weinberg RA. Estrogen-dependent cyclin E-cdk2 activation through p21 redistribution. Mol Cell Biol. 1997; 17(7): 4059–4069. (doi:10.1128/MCB.17.7.4059)

47. Foster JS, Henley DC, Ahamed S, Wimalasena J. Estrogens and cell-cycle regulation in breast cancer. Trends Endocrinol Metab. 2001; 12(7): 320–327. (doi:10.1016/S1043-2760(01)00436-2)

48. Prall OWJ, Sarcevic B, Musgrove EA, Watts CKW, Sutherland RL. Estrogen-induced activation of Cdk4 and Cdk2 during G1-S phase progression is accompanied by increased cyclin D1 expression and decreased cyclin-dependent kinase inhibitor association with cyclin E-Cdk2. J Biol Chem. 1997; 272(16): 10882–10894. (doi:10.1074/jbc.272.16.10882)

49. Carroll JS, Prall OWJ, Musgrove EA, Sutherland RL. A pure estrogen antagonist inhibits cyclin E-Cdk2 activity in MCF-7 breast cancer cells and induces accumulation of p130-E2F4 complexes characteristic of quiescence. J Biol Chem. 2000; 275(49): 38221–38229. (doi:10.1074/jbc.M004424200)

50. Watts CK, Brady A, Sarcevic B, deFazio A, Musgrove EA, Sutherland RL. Antiestrogen inhibition of cell cycle progression in breast cancer cells in associated with inhibition of cyclin-dependent kinase activity and decreased retinoblastoma protein phosphorylation. Mol Endocrinol. 1995; 9(12): 1804–1813. (doi:10.1210/mend.9.12.8614416)

51. Cicatiello L, Addeo R, Altucci L, Belsito Petrizzi V, Boccia V, Cancemi M, et al. The antiestrogen ICI 182,780 inhibits proliferation of human breast cancer cells by interfering with multiple, sequential estrogen-regulated processes required for cell cycle completion. Mol Cell Endocrinol. 2000; 165(1–2): 199–209. (doi:10.1016/S0303-7207(00)00243-4)

52. Truchet I, Jozan S, Guerrin M, Mazzolini L, Vidal S, Valette A. Interconnections between E2-dependent regulation of cell cycle progression and apoptosis in MCF-7 tumors growing on nude mice. Exp Cell Res. 2000; 254(2): 241–248. (doi:10.1006/excr.1999.4756)

53. Doisneau-Sixou SF, Sergio CM, Carroll JS, Hui R, Musgrove EA, Sutherland RL. Estrogen and antiestrogen regulation of cell cycle progression in breast cancer cells. Endocr Relat Cancer. 2003; 10(2): 179–186. (doi:10.1677/erc.0.0100179)

54. Giacinti C, Giordano A. RB and cell cycle progression. Oncogene. 2006; 25(38): 5220–5227. (doi:10.1038/sj.onc.1209615)

55. Dannenberg J, Rossum A Van, Schuijff L, Dannenberg J, Rossum A Van, Schuijff L, et al. Ablation of the Retinoblastoma gene family deregulates G 1 control causing immortalization and increased cell turnover under growth-restricting conditions. Genes Dev. 2000; 3051–3064. (doi:10.1101/gad.847700)

56. Sherr CJ, Roberts JM. PERSPECTIVE CDK inhibitors: positive and negative regulators of G_1-phase progression. 1999;(901): 1501–1512. (doi:10.1101/gad.13.12.1501)

57. Bockstaele L, Coulonval K, Kooken H, Paternot S, Roger PP. Regulation of CDK4. Cell Div. 2006; 1:1–16. (doi:10.1186/1747-1028-1-25)

58. Olashaw N, Bagui TK, Pledger WJ. Cell cycle control: a complex issue. Cell Cycle. 2004 Mar; 3(3): 263–264. (doi:10.4161/cc.3.3.720)

59. Perez-Roger I, Solomon DL, Sewing A, Land H. Myc activation of cyclin E/Cdk2 kinase involves induction of cyclin E gene transcription and inhibition of p27 (Kip1) binding to newly formed complexes. Oncogene. 1997 May; 14(20): 2373–2381. (doi:10.1038/sj.onc.1201197)

60. Watts CKW, Sweeney KJE, Warlters A, Musgrove EA, Sutherland RL. Antiestrogen regulation of cell cycle progression and cyclin D1 gene expression in MCF-7 human breast cancer cells. Breast Cancer Res Treat. 1994; 31(1): 95–105. (doi:10.1007/BF00689680)

61. Gill RM, Hamel PA, Zhe J, Zacksenhaus E, Gallie BL, Phillips RA. Characterization of the human RB1 promoter and of elements involved in transcriptional regulation. Cell Growth Differ. 1994; 5(5): 467–474.

62. Ohtani, K., DeGregori, J. and Nevins, J. R. (1995) Regulation of the cyclin E gene by transcription factor E2F1. Proc. Natl. Acad. Sci. U.S.A. 92, 12146-12150. (doi: 10.1073/pnas.92.26.12146)

63. Thalmeier K, Synovzik H, Mertz R, Winnacker EL, Lipp M. Nuclear factor E2F mediates basic transcription and trans-activation by E1a of the human MYC promoter. Genes Dev. 1989 Apr; 3(4): 527–536. (doi:10.1101/gad.3.4.527)

64. Carroll JS, Swarbrick A, Musgrove EA, Sutherland RL. Mechanisms of growth arrest by c-myc antisense oligonucleotides in MCF-7 breast cancer cells: implications for the antiproliferative effects of antiestrogens. Cancer Res. 2002; 62(11): 3126–3131.

65. Thiantanawat A, Long BJ, Brodie AM, Thiantanawat A, Long BJ, Brodie AM. Signaling Pathways of Apoptosis Activated by Aromatase Inhibitors. Cancer Res. 2003; 8037–8050.

66. Wijayaratne AL, McDonnell DP. The Human Estrogen Receptor-α is a Ubiquitinated Protein Whose Stability is Affected Differentially by Agonists, Antagonists, and Selective Estrogen Receptor Modulators. J Biol Chem. 2001; 276(38): 35684–35692. (doi:10.1074/jbc.M101097200)

67. Alao JP. The regulation of cyclin D1 degradation: roles in cancer development and the potential for therapeutic invention. Mol Cancer. 2007 Apr; 6:24. (doi:10.1186/1476-4598-6-24)

68. Gregory MA, Hann SR. c-Myc proteolysis by the ubiquitin-proteasome pathway: stabilization of c-Myc in Burkitt's lymphoma cells. Mol Cell Biol. 2000 Apr; 20(7): 2423–2435. (doi:10.1128/MCB.20.7.2423-2435.2000)

69. Abbas T, Dutta A. p21 in cancer: intricate networks and multiple activities. Nat Rev Cancer. 2009 Jun; 9(6): 400–414. (doi:10.1038/nrc2657)

70. Singer JD, Gurian-West M, Clurman B, Roberts JM. Cullin-3 targets cyclin E for ubiquitination and controls S phase in mammalian cells. Genes Dev. 1999 Sep; 13(18): 2375–2387. (doi:10.1101/gad.13.18.2375)

71. Oh K-J, Kalinina A, Bagchi S. Destabilization of Rb by human papillomavirus E7 is cell cycle dependent: E2-25K is involved in the proteolysis. Virology. 2010 Jan; 396(1): 118–124. (doi:10.1016/j.virol.2009.10.018)

72. Martinez-Chavez A, Rosing H, Hillebrand M, Tibben M, Schinkel AH, Beijnen JH. Development and validation of a bioanalytical method for the quantification of the CDK4/6 inhibitors abemaciclib, palbociclib, and ribociclib in human and mouse matrices using liquid chromatography-tandem mass spectrometry. Anal Bioanal Chem. 2019 Aug;411(20):5331–45. (doi: 10.1007/s00216-019-01932-w)

73. Alegete P, Kancherla P, Albaseer S. S, Boodida S. A Validated Liquid Chromatography–Tandem Mass Spectrometric (LC-MS/MS) Method for the Estimation of Fulvestrant in Human Plasma. Orient J Chem 2017;33(3). (doi: 10.13005/ojc/330312)

74. Jr RR. Cyclin-dependent protein kinase inhibitors including palbociclib as anticancer drugs. Pharmacol Res. Elsevier Ltd; 2016; 107:249–275. (doi:10.1016/j.phrs.2016.03.012)

75. Classon M, Harlow E. The retinoblastoma tumour suppressor in development and cancer. Nat Rev Cancer. 2002; 2(12):910–917. (doi:10.1038/nrc950)

76. MacDonald JI, Dick FA. Posttranslational modifications of the retinoblastoma tumor suppressor protein as determinants of function. Genes and Cancer. 2012; 3(11–12): 619–633. (doi:10.1177/1947601912473305)

77. Lents NH, Gorges LL, Baldassare JJ. Reverse mutational analysis reveals threonine-373 as a potentially sufficient phosphorylation site for inactivation of the retinoblastoma tumor suppressor protein (pRB). Cell Cycle. 2006; 5(15): 1699–1707. (doi:10.4161/cc.5.15.3126)

78. Narasimha AM, Kaulich M, Shapiro GS, Choi YJ, Sicinski P, Dowdy SF. Cyclin D activates the Rb tumor suppressor by mono-phosphorylation. Elife. 2014; 3:1–21. (doi:10.7554/eLife.02872)

79. Zarkowska T, Mittnacht S. Differential Phosphorylation of the Retinoblastoma Protein by G1/S Cyclin-dependent Kinases*. Biochemistry. 1997; 272(19): 12738–12746. (doi:10.1074/jbc.272.19.12738)

80. Gladden AB, Diehl JA. Cell cycle progression without cyclin E/CDK2: Breaking down the walls of dogma. Cancer Cell. 2003; 4(3): 160–162. (doi:10.1016/S1535-6108(03)00217-4)

81. Chassot A-A, Lossaint G, Turchi L, Meneguzzi G, Fisher D, Ponzio G, et al. Confluence-induced cell cycle exit involves pre-mitotic CDK inhibition by p27(Kip1) and cyclin D1 downregulation. Cell Cycle. 2008 Jul; 7(13): 2038–2046. (doi:10.4161/cc.7.13.6233)

82. Hannan KM, Kennedy BK, Cavanaugh AH, Hannan RD, Hirschler-Laszkiewicz I, Jefferson LS, et al. RNA polymerase I transcription in confluent cells: Rb downregulates rDNA transcription during confluence-induced cell cycle arrest. Oncogene. 2000 Jul 21; 19:3487. (doi:10.1038/sj.onc.1203690)

83. Viñals F, Pouysségur J. Confluence of Vascular Endothelial Cells Induces Cell Cycle Exit by Inhibiting p42/p44 Mitogen-Activated Protein Kinase Activity. Mol Cell Biol. 1999 Apr 1; 19(4): 2763 LP-2772. (doi:10.1128/MCB.19.4.2763)

84. Wright Muelas M, Ortega F, Breitling R, Bendtsen C, Westerhoff H V. Rational cell culture optimization enhances experimental reproducibility in cancer cells. Sci Rep. 2018;8(1): 1–16. (doi:10.1038/s41598-018-21050-4)

85. Zi Z. Sensitivity analysis approaches applied to systems biology models. IET Syst Biol. 2011; 5(6): 336–346. (doi:10.1049/iet-syb.2011.0015)

86. Nagaraja S, Wallqvist A, Reifman J, Mitrophanov AY. Computational Approach To Characterize Causative Factors and Molecular Indicators of Chronic Wound Inflammation. J Immunol. 2014; 192(4):1824–1834. (doi:10.4049/jimmunol.1302481)

87. He L, Kulesskiy E, Saarela J, Turunen L, Wennerberg K, Aittokallio T, Tang J. "Methods for High-Throughput Drug Combination Screening and Synergy Scoring." In von Stechow, Louise (eds.), Cancer Systems Biology: Methods and Protocols, chapter 17, 351-398. Springer New York. (doi: 10.1007/978-1-4939-7493-1_17)

88. Finn RS, Crown JP, Lang I, Boer K, Bondarenko IM, Kulyk SO, Ettl J, Patel R, Pinter T, Schmidt. M, et al. The cyclin-dependent kinase 4/6 inhibitor palbociclib in combination with letrozole versus letrozole alone as first-line treatment of oestrogen receptor-positive, HER2-negative,

advanced breast cancer (PALOMA-1/TRIO-18): a randomised phase 2 study. Lancet Oncol. England; 2015 Jan; 16(1): 25–35. (doi:10.1016/S1470-2045(14)71159-3)

89. Cristofanilli M, Turner NC, Bondarenko I, Ro J, Im S-A, Masuda N, Colleoni M, DeMichele A, Loi, S, Verma S, et al. Fulvestrant plus palbociclib versus fulvestrant plus placebo for treatment of hormone-receptor-positive, HER2-negative metastatic breast cancer that progressed on previous endocrine therapy (PALOMA-3): final analysis of the multicentre, double-blind, phase 3 randomised controlled trial. Lancet Oncol. England; 2016; 17(4): 425–439. (doi:10.1016/S1470-2045(15)00613-0)

90. Stearns V, Brufsky AM, Verma S, Cotter MJ, Lu DR, Dequen F, Joy AA. Expanded-Access Study of Palbociclib in Combination With Letrozole for Treatment of Postmenopausal Women With Hormone Receptor–Positive, HER2-Negative Advanced Breast Cancer. Clin Breast Cancer. 2018; 18(6): e1239–e1245. (doi:10.1016/j.clbc.2018.07.007)
